# A *Biomphalaria glabrata* peptide that stimulates significant behaviour modifications in aquatic free-living *Schistosoma mansoni* miracidia

**DOI:** 10.1371/journal.pntd.0006948

**Published:** 2019-01-22

**Authors:** Tianfang Wang, Russell C. Wyeth, Di Liang, Utpal Bose, Guoying Ni, Donald P. McManus, Scott F. Cummins

**Affiliations:** 1 School of Science and Education, Genecology Research Centre, University of the Sunshine Coast, Maroochydore DC, Queensland, Australia; 2 Department of Biology, St. Francis Xavier University, Antigonish, Nova Scotia, Canada; 3 School of Medical Science, Griffith Health Institute, Griffith University, Gold Coast, Queensland, Australia; 4 QIMR Berghofer Medical Research Institute, Brisbane, Queensland, Australia; Center for Discovery and Innovation in Parasitic Diseases, UNITED STATES

## Abstract

The human disease schistosomiasis (or bilharzia) is caused by the helminth blood fluke parasite *Schistosoma mansoni*, which requires an intermediate host, the freshwater gastropod snail *Biomphalaria glabrata* (the most common intermediate host). The free-swimming parasite miracidia utilise an excellent chemosensory sense to detect and locate an appropriate host. This study investigated the biomolecules released by the snail that stimulate changes in the behaviour of the aquatic *S*. *mansoni* miracidia. To achieve this, we have performed an integrated analysis of the snail-conditioned water, through chromatography and bioassay-guided behaviour observations, followed by mass spectrometry. A single fraction containing multiple putative peptides could stimulate extreme swimming behaviour modifications (e.g. velocity, angular variation) similar to those observed in response to crude snail mucus. One peptide (P12;—R-*DITSGLDPEVADD*-KR—) could replicate the stimulation of miracidia behaviour changes. P12 is derived from a larger precursor protein with a signal peptide and multiple dibasic cleavage sites, which is synthesised in various tissues of the snail, including the central nervous system and foot. P12 consists of an alpha helix secondary structure as indicated by circular dichroism spectroscopy. This information will be helpful for the development of approaches to manipulate this parasites life cycle, and opens up new avenues for exploring other parasitic diseases which have an aquatic phase using methods detailed in this investigation.

## Author summary

In aquatic environments, where the vast majority of animals live in darkness, key relationships are often formed and maintained by chemical communication (including smell and taste). Parasites with an aquatic life phase rely on an exquisite sense of chemosensation to detect host biomolecules (kairomones), allowing them to locate and infect their host. Our study identifies the first kairomone released by the freshwater gastropod snail *Biomphalaria glabrata*, an intermediate host for the helminth blood fluke parasite *Schistosoma mansoni*. This is a key aspect of the *S*. *mansoni* life-cycle that ultimately leads to human infection, causing the disease schistosomiasis (or bilharzia), which is considered the most devastating human helminth infection in terms of global morbidity and mortality. The kairomone we identify is a peptide that does not appear to share any similarity with any other known animal peptide. This information will be helpful as we explore methods to interrupt parasite infection, and therefore break the cycle of infection that causes a major human disease.

## Introduction

Worldwide, an estimated 200 million people are infected and over 800 million people are at risk of infection of Schistosomiasis [1–3], while over 200,000 people yearly will die from schistosomiasis-related illness [4]. This disease also has a high morbidity rate, being responsible for the loss of up to 4.5 million disability adjusted life years annually[5], thus providing strong humanitarian and economic incentive to conduct research on various aspects of the epidemiology and ecology of the disease [6, 7]. Though the widespread drug treatment (praziquantel, PZQ) has decreased disease prevalence, schistosomiasis endemicity has remained stable due principally to poor sanitary conditions that give rise to constant re-infection [8–10]. PZQ helps control disease through decreasing worm burden and thus transmission of the parasite into the environment, however, it is not sufficient to eliminate the disease [11]. There is also the potential for the emergence of drug-resistant parasites [12, 13].

The co-evolutionary dynamics of the host-parasite interaction take the form of a fierce arms race, where evolutionary drive rapidly alters facets of the interaction to best suit shifting environmental circumstances [14]. With that, the life-cycle of *S*. *mansoni* is complex, involving two critical infection stages, where i) aquatic miracidia infect a snail intermediate host, e.g., *Biomphalaria glabrata*, and ii) aquatic cercariae infect a primary host, principally humans (or other mammals). Research efforts have predominantly studied the second stage, and many countries invest in intervention strategies based on short-term control programs involving mass drug administration that do not address the parasite reservoir [15, 16, 17]. Long-term solutions could be forthcoming through more in-depth investigation of the first stage, where schistosome miracidia locate and infect their snail hosts after egg hatching.

In the natural environment, miracidia will modify their behaviour upon detection of attractive biomolecule(s), characterised by increased rate of change of direction (RCD). Kairomones are chemicals emitted by a species that are detected by another species, whereby only the detecting species benefits. Experiments have been performed to study the attractant biomolecule(s) used by *S*. *mansoni* miracidia for locating *B*. *glabrata*, primarily through microsystems offering snail-conditioned water (SCW) at concentrations that may be inconsistent with the natural environment [18]. The relevant behaviours of miracidia have also been studied and modelled in with the presence of SCW, that angular velocity of miracidia increased by 3 times in concentration gradients of SCW [19] and significant accumulation (more than 60%) of miracidia was observed in a spot of SCW in an artificial pond [20]. Non-specific small molecular weight biomolecules present within SCW were considered to contain the attractant, and this was supported by experimental assays showing an increase in miracidia RCD and turn-back responses [21]. Further, macromolecular glycoconjugates within SCW, referred to as miracidia-attracting glycoconjugates (MAGs), have been implicated following an observed induction of changes to miracidial RCD and turn-back responses [19, 22, 23]. Other studies confirm the existence of parasite attractants in SCW derived from other species; for example, miracidia of *Trichobilharzia ocellata* and *Trichobilharzia franki*, but these species respond only to SCW from their particular host-snail species. An interesting point to note is that while species-specificity was found in the Egyptian strain of *S*. *mansoni*, the *S*. *mansoni* strain from Brazil was found to respond to SCW from host as well as from some non-host-snail species. This degree of non-specificity has also been observed in the host-finding behaviour of another trematode parasite, *Echinostoma caproni* [18, 24–26]. These findings implicate a multi-component blend of biomolecules as potential kairomones, both general and host-specific.

Towards identifying the kairomone biomolecule(s) that helps *S*. *mansoni* miracidia detect and locate its host, we have performed an integrated analysis of the SCW, through chromatography and bioassay-guided behaviour observations, followed by mass spectrometry.

## Methods

### Ethics statement

The conduct and procedures involving animal experimentation were approved by the Animal Ethics Committee of the QIMR Berghofer Medical Research Institute, Brisbane (Project number P242). This study was performed in accordance with the recommendations in the Guide for the Care and Use of Laboratory Animals of the National Institutes of Health.

### Collection of snail-conditioned water (SCW)

**Fig 1:**
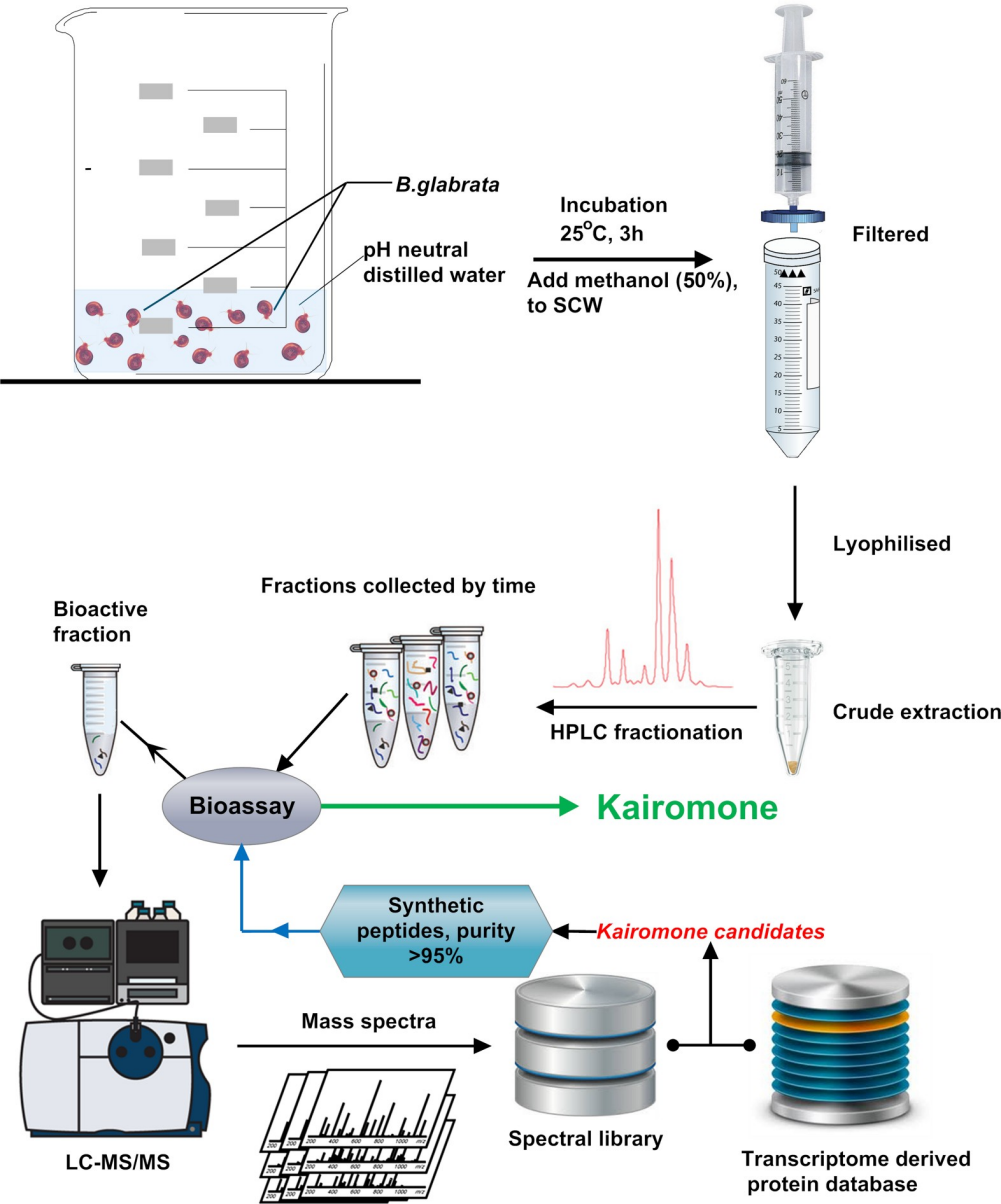
Workflow for identification of metabolites and peptides within *B*. *glabrata* conditioned water, then active biomolecule identification. Snail conditioned water (SCW) was fractionated using HPLC followed by bioassay and identification using nanoLC tandem TripleTOF MS/MS.

The samples were collected at the snail facility at QIMR Berghofer Medical Research Institute; multiple 20 snail batches (*B*. *glabrata*, BB02 strain) were collected from different aquaria, washed thoroughly with pH neutral MilliQ water, and kept in beakers holding 20 snails in approximately 25 ml water for 3 h at 28°C as shown in **Fig 1**. Following this incubation, snails were returned to their original aquaria, and 25 ml methanol was added into each beaker of SCW and mixed thoroughly, the mixture was filtered through 0.45 μm PVDF Millex-HV syringe filter units to remove particles and microbes. The filtrate was snapped frozen and lyophilised. As negative controls, SCW from the land snail *Theba pisana* and tropical freshwater snail *Oncomelania quadrasi* were also collected, filtered, lyophilised and subjected to bioassay. Raw mucus was also collected directly from the beaker without any process as a positive control.

### Isolation of *S*. *mansoni* miracidia

The Puerto Rican strain of *S*. *mansoni* was maintained, under permit from the Australian Department Agriculture, Fisheries and Forestry Biosecurity (DAFF), in ARC Swiss mice and *B*. *glabrata* snails at QIMR-B from stock originating from the National Institute of Allergy and Infectious Diseases Schistosomiasis Resource Centre, Biomedical Research Institute (Rockville, Maryland, USA).

Mice were euthanised with CO_2_ gas and their livers were perfused with chilled phosphate buffered saline (PBS). Eggs of *S*. *mansoni* were collected during perfusion of mice. Four infected mouse livers were sliced with scalpel blades and blended to a smooth consistency in 50 ml PBS. The mixture was centrifuged (2,000g at 4°C for 10 s), then supernatant was removed and pellet re-suspended in 50 ml chilled PBS. The wash step was repeated four times until the supernatant was relatively clear and transparent. The pellet (containing liver tissue and eggs) was finally reconstituted in pH neutral spring water. Miracidia were hatched from eggs at 28°C and collected 2 h post-hatch (hph) under an inverted light microscope using a Pasteur pipette.

### Protein fractionation using HPLC

The lyophilised SCW crude extract was resuspended in 0.1% trifluoroacetic acid (TFA) and centrifuged at 16,000 g for 20 mins at 4°C. The supernatant was collected and injected into the high-performance liquid chromatography (HPLC, PerkinElmer, USA) equipped with a 250 mm × 4.6 μm ZORBAX SB-C18 column (Agilent Technologies, Australia). Linear gradients of 0–60% solvent B over 60 min at 0.5 ml/min flow rate, followed by a steeper gradient from 60% to 80% solvent B in 5 min were used for peptide elution. Solvent B was increased to 95% in 5min and held at 95% for 5 min to wash the column. Solvent A consisted of 0.1% TFA in MQ water and solvent B contained 0.1% TFA in 100% acetonitrile. The eluted fractions were collected every 4 min, lyophilised and subjected to bioassay.

### UHPLC-QToF-MS analysis of metabolites

Samples were analysed using an Agilent UHPLC-Q-ToF-MS system comprising a 1290 UHPLC coupled to a 6520 Accurate-Mass Quadrupole Time-of-Flight Mass Spectrometer (QToF-MS) in fast polarity switching mode from *m/z* 100 to 1700 for all samples at a scan rate of 3 cycles. Instrument resolution was 9000–11700 across the data acquisition range. This mass range enabled the inclusion of two reference compounds, a lock mass solution including purine (C_5_H_4_N_4_ at *m/z* 121.050873, 10 μmol/l) and hexakis (1H, 1H, 3H-tetrafluropentoxy)-phosphazene (C_18_H_18_O_6_N_3_P_3_F_24_ at m/z 922.009798, 2 μmol). Chromatographic separation was achieved using an Agilent Poroshell UHPLC column (150 mm x 4.6 mm, 2.7 μm). The mobile phase consisted of (A) MilliQ water with (B) acetonitrile (ACN) (LabScan Analytical Science, Australia). In all HPLC runs the elution gradient started at 80% A: 20% B increasing to 0% A: 100% B over 30 min, followed by a 3 min hold and 12 minutes re-equilibration period. Data was acquired in positive and negative mode. A sample volume of 20 μl was injected for each HPLC run. The HPLC run contained blanks, a sample-relevant standard solution and pooled samples intercalated throughout the HPLC run to control for any acquisition-dependent variation. The samples and standards were filtered using a 0.2 μm PTFE membrane filter (Phenomenex, Torrance, CA, USA) before analysis.

Multimode (Electrospray Ionization (ESI) and Atmospheric Pressure Chemical Ionization (APCI)) with Fast Polarity Switching (FPS) was used to ionise and detect compounds after chromatographic separation. The general parameters of the MS1 mixed mode source were as follows: capillary voltage 3500 V, nebulizer pressure 30 psi, drying gas 7.0 l/min, gas temperature 300°C, vaporizer 200 V, voltage charge 2000 V; negative-ion mode capillary voltage 2500 V, corona negative 15.0 V, fragmentor 175 V, skimmer1 65.0 V, octopole RF Peak 750 V, positive ion mode capillary voltage 2500 V, corona positive 4.0 V, fragmentor 175 V, skimmer1 65.0 V, and octopole RF Peak 750 V. Data processing was performed using Agilent MassHunter Qualitative software (Version B.05.00).

### Metabolite identification

Data analysis was performed using Agilent MassHunter Qualitative software (Version B.05.00), referring to METLIN [27] and HMDB [28] databases for mass spectra searches. The Molecular Feature Extractor (MFE) algorithm within MassHunter Qualitative analysis software was used to extract chemically qualified molecular features from the LC-QToF-MS data files. This algorithm uses a wide range of MS information, including accurate mass measurements, adduct formation, multimer formation and isotope patterns to generate a list of candidate compounds. For empirical formula generation, the Molecular Formula Generator (MFG) algorithm was used [29]. The maximum elemental composition C_60_H_120_O_30_N_30_S_5_Cl_3_Br_3_ was used to generate formulae.

### NanoHPLC-ESI-Triple TOF analysis of proteins

Three fractions that indicated bioactivity in bioassay (see Bioassay and peptide synthesis), were analysed by LC-MS/MS on a Shimadzu Prominance Nano HPLC (Japan) coupled to a Triple Tof 5600 mass spectrometer (ABSCIEX, Canada) equipped with a nano electrospray ion source, following the method described elsewhere [30]. Briefly, 6 μl of re-suspended solution was injected onto a C18 trap column (Agilent Technologies, Australia). The sample was de-salted on the trap column, which was placed in-line with the analytical nano HPLC column. Linear gradients of 1–40% solvent B over 30 min at 300 nl/min flow rate, followed by a steeper gradient from 40% to 80% solvent B in 5 min were used for peptide elution. Solvent B was held at 80% for 5 min to wash the column and returned to 100% solvent A for equilibration prior to the next sample injection. Solvent A consisted of 0.1% formic acid (aq) and solvent B contained 90/10 acetonitrile/0.1% formic acid (aq). The ion spray voltage was set to 2400V, declustering potential (DP) 100V, curtain gas flow 25, nebuliser gas 1 (GS1) 12 and interface heater at 150°C. Full scan TOFMS data was acquired over the mass range 200–1800 and for product ion ms/ms in the range 100–1800. Ions observed in the TOF-MS scan exceeding a threshold of 100 counts and a charge state of +2 to +5 were set to trigger the acquisition of product ion. The data were acquired and processed using Analyst TF 1.5.1 software (ABSCIEX, Concord, Canada).

### Protein identification and gene expression

*B*. *glabrata* transcriptome files were downloaded from the *Biomphalaria* genome consortium server (http://genome.wustl.edu/genomes/detail/biomphalaria-glabrata/), from which open reading frames were predicted by CLC workbench and used for constructing the protein database utilised in mass spectral analysis. A composite target decoy database was built with the forward and reverse sequences for calculating the false discovery rate. Proteins were identified by database searching using PEAKS v7.0 (Bioinformatics Solutions Inc., Waterloo, ON, Canada) against the protein database (**Fig 1**). Search parameters were as follows: fully tryptic enzyme specificity with no digestion, variable modifications included amidation, methionine oxidation, conversion of glutamine to pyroglutamic acid, and deamidation of asparagine. Precursor mass error tolerance was set to 20 ppm and a fragment ion mass error tolerance was set to 0.1 Da; the false discovery rate was set to ≤1%, and the individual peptide ion score [-10*Log(p)] was calculated accordingly, where p is the probability that the observed match is a random event. Identified proteins were subject to BLASTp and tBLASTn using the corresponding database of NCBI. Protein N-terminal signal sequences were predicted using the SignalP 4.1 [31]. Proteolytic cleavage sites, as well as post-translational modifications, were predicted based on homology to other known peptides and the Neuropred tool (neuroproteomics.scs.illinois.edu/neuropred.html).

High-quality clean RNA-seq reads from *B*. *glabrata* tissues (albumen gland, buccal mass, central nervous system, digestive gland, foot, heart/amebocyte-producing organ, kidney mantle salivary glands, stomach and terminal genitalia) were obtained from Vectorbase (https://www.vectorbase.org/organisms/biomphalaria-glabrata), then mapped to the genome scaffolds using the CLC Genomic Workbench 9 software (CLC Bio-Qiagen, Aarhus, Denmark). Relative expression of genes in the transcriptome was determined based on transcripts per kilobase million mapped reads (TPM) values, utilizing the *de novo* RNA-seq CLC Genomic Workbench 9 software.

### Miracidia behaviour in response to test solutions

Test solutions: SCW crude extracts from *B*. *glabrata*, *T*. *pisana* and *O*. *quadrasi*, HPLC-eluted fractions (SCW of *B*. *glabrata*). Selected peptides identified from the bioactive fraction were synthesised by GenicBio Biotech (Hongkong, China) to a purity >95%. Each peptide was initially dissolved in pH neutral spring water to 1 mg/ml, which was then diluted to 0.1 and 0.01 mg/ml for bioassays. As negative controls, MilliQ water was subjected to bioassay.

**Fig 2:**
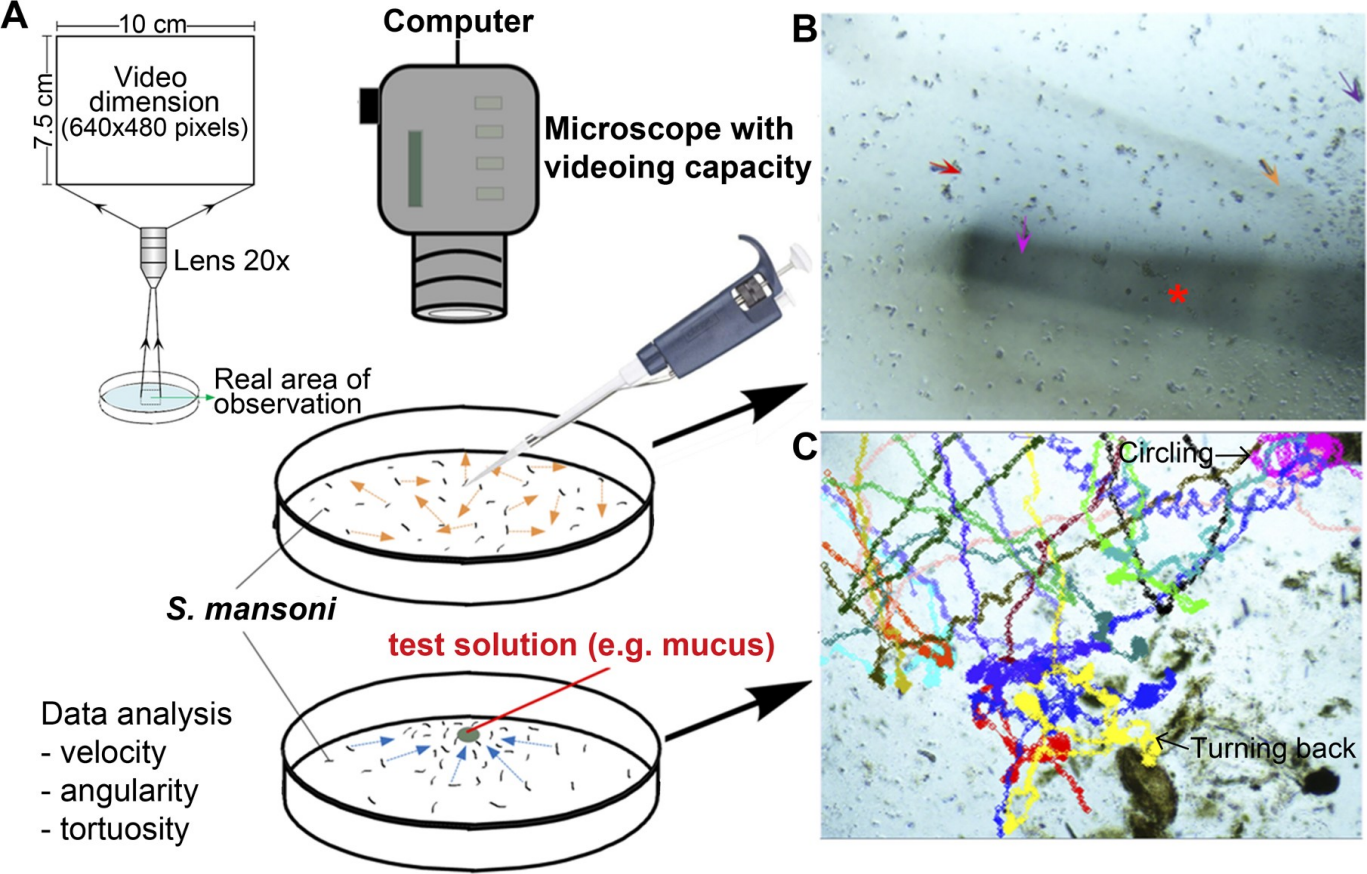
Characterization of the behaviour of *S*. *mansoni* miracidia in response to raw mucus of *B*. *glabrata*. (**A**) Arrangement of bioassay, including schematic illustration of video zone and corresponding area of observation. (**B**) Microscopic examination of site of application. Arrows show sites of miracidia for subsequent tracking. Asterisk indicates the pipette tip shadow. (**C**) Trajectories of miracidia in the presence of raw mucus. Different colours represent individual miracidium.

Miracidia collection and assay: *S*. *mansoni* miracidia at ~2 hph were used for each assay, 30±5 actively swimming miracidia in spring water with a pH of 7.0 (~4 ml in total) were evenly distributed with a pipette to the central region of a Petri dish (100 mm × 15 mm) containing 4 ml of spring water (**Fig 2A**). For each fraction/pure peptide, bioassays were repeated three times; in addition, bioassays were performed in triplicate for P12 with different concentrations. The swimming area for the miracidia was covered to prevent light bias prior to analysis under the microscope. In assays, 2 μl of test solution was added to the central area of the Petri dish (**Fig 2B**). Some diffusion of the molecule is expected over the 1 min test period. Assays were also tested at 10×, 100× and 1000x serial dilutions. To record miracidia movement before and after addition of test solutions, an inverted compound microscope with videoing capacity (OLYMPUS CKX41) fitted with an OLMPUS DPI Digital Microscope Camera DP22 (2.8 megapixel image at a rate of 25 frames per second) was used. The real camera’s field of view (FOV) was 2.500×1.875 mm. Miracidia movement was recorded for 1 min before and after the addition of the test solutions, then captured videos were processed using Tracker 4.87 (https://physlets.org/tracker/). All bioassays were carried out on a batch of minimum biological triplicates, with one video presented as S1–S10 Movies.

Analysis: Initially, miracidia trajectories were tracked manually from entrance into the FOV to exit, or up to 1 min for those that remained within the FOV (**Fig 2C**). At both, before and after solution addition, only miracidia that had been swimming for more than the length of the short edge (7.5 cm) of the FOV were included. The average time duration of miracidia staying within the FOV was considered as another key behavioural feature and was statistically compared. For those miracidia staying for more than 1 min after addition of solution, the time duration within that 1 min was used for comparison, and the mean acceleration value was calculated. Miracidium acceleration and velocity were calculated based on trajectories, and with units converted to cm s^-2^ and cm s^-1^. A paired two-tailed t-test was used to calculate P-values. To fully evaluate behavioural changes across serial dilutions, a more detailed analysis of miracidia tracks was conducted. First, contrast was enhanced by a rolling mean subtraction [32], after which the TrackMate plugin [33] for FIJI software [34] was used to detect individual miracidia in each frame, and then link frame-by-frame x,y locations into tracks. Manual verification of a subset of tracks verified this semi-automated approached accurately tracked multiple miracidia simultaneously through the videos. Track data were then imported into a second FIJI plugin, MTrackJ [35], facilitating calculation of three individual behavioural measurements: speed, angular standard deviation (a measure of the magnitude and frequency of turns), and tortuosity (the ratio of track length to maximum displacement, ranging from one [a straight line] to infinity [a purely theoretical set of all possible lines that each curve to fill the entire plane before reaching their end-point]), as well as the group measurement of the number of miracidia present in view per unit time. Averages were calculated for each measurement for each trial, and these trial-level data were then used for statistical analyses comparing measurements before and after addition of treatment solutions. Paired t-tests of these pooled samples showed that although small changes in speed and turning rate did occur (either as a non-specific response to peptide solutions or due to a consistent time-dependent change in behaviour irrespective of any addition of solutions), there were no significant changes in the overall tortuosity of track nor the rate at which miracidia aggregated in the field of view. Thus, we concluded our application protocol and the non-specific addition of a peptide did not cause substantial changes in behaviour, and we therefore subsequently tested the effects of various peptides using this procedure.

### Circular dichroism (CD) spectroscopy

CD experiments were performed in a Chirascan CD spectrophotometer (Applied Photophysics, Leatherhead, UK). A quartz cuvette with a 10 mm path length was used for the recording of spectra over a wavelength range of 190–260 nm with a 1 nm bandwidth, 1 nm step size and time of 0.5 s per point. A buffer baseline was collected in the same cuvette and was subtracted from the sample spectra.

### Structure prediction of P12 peptide using replica-exchange molecular dynamics (REMD) simulation

Protein secondary structure predictions were made using the Assisted Model Building with Energy Refinement (AMBER) 14 [36] with force field parameters ff14SB. REMD [37] simulation method incorporated in SANDER module of AMBER was used. The generalised Born/solvent-accessible surface area (GB/SA) implicit solvent model [Bondi radii, solvent dielectric constant 78.5, surface tension 0.005 cal·mol^–1^·Å^2^] [38] was used to model the effects of solvation [39]. All intra-peptide non-bonded interactions were included in the calculation. The SHAKE algorithm [40] with a relative geometric tolerance of 10^−5^ was used to constrain all bond lengths to their equilibrium distances, and a 2-fs time step was used.

The P12 peptide was initially built in extended conformations. It was then subjected to 1,500 steps of steepest descent minimization, followed by a single equilibration trajectory of 40 ps where the temperature was established by velocity reassignment from a Maxwell–Boltzmann distribution at 325 K and maintained at that temperature by using a Berendsen thermostat [41] with a coupling constant of 1 ps^–1^. The final state of this simulation was used as the initial conformation for the subsequent REMD.

The REMD was implemented in SANDER of AMBER. Twelve replicas were simulated over a range of temperatures from 269.5 to 450.0 K: 269.5, 281.3, 293.5, 306.4, 319.7, 333.7, 348.2, 363.4, 379.3, 395.9, 413.1, 431.2 and 450.0 K. The number of replicas was determined based on the total number of atoms of peptide. Exchange attempts were made after every 2 ps of simulation. Average acceptance ratio for the replica-swap is from 30% to 80%. A Maxwell–Boltzmann distribution (after each exchange attempt) in conjunction with a Berendsen thermostat [41] with a coupling constant of 1 ps^–1^ was used to maintain the replica temperatures. An initial replica-exchange equilibration period of 200 ps per replica was carried out, followed by REMD of each replica every 0.2 ps over a production simulation time of 200 ns. This yielded a total of 100,000 conformations at each temperature. The structural information P12 at 306.4 K (a temperature close to the temperature of the environment in which *B*. *glabrata* and miracidia exist) was extracted for secondary structure calculation and analysis.

## Results

### The behaviours of *S*. *mansoni* miracidia in the presence of raw or filtered *B*. *glabrata* mucus

We first determined whether raw mucus from *B*. *glabrata* (strain BB02) could stimulate behaviour changes in *S*. *mansoni* (Puerto Rican strain) miracidia. Using *in vivo* bioassay, qualitative observations showed changes in acceleration, velocity and turning following the addition of snail crude mucus (**S1 Movie**). After an indefinite period of post-exposure, miracidia localised their movement to strictly within the mucus application zone, and exhibited a noticeable movement involving repeated extension and contraction, as well as periods of rapid rotation from their anterior point (**S1 Movie**).

We additionally observed that snail crude mucus and SCW that had been filtered through a 0.45 μm filter retained the bioactive properties attributed to the proposed biomolecule(s) (**S2 Movie**). As negative controls, crude mucus extracts from the freshwater snail *Oncomelania hupensis quadrasi* (intermediate host of *Schistosoma japonicum*, but not of *S*. *mansoni*) and the land snail *Theba pisana* (non-*Schistosoma* host) were tested on *S*. *mansomi* miracidia. No behaviour changes were observed with either of these SCW extracts (**S1 Fig and S3 and S4 Movies**).

### The determination of HPLC fraction altering miracidium behaviours

To determine the identity of the *Biomphalaria*-derived biomolecule(s) (kairomones) that stimulate behaviour changes in *S*. *mansoni* miracidia, we first carried out a proteomic analysis of the 0.45 μm-filtered SCW, following the procedure described in **Fig 1**. Briefly, lyophilised crude extracts from SCW were HPLC-fractionated for bioassays to assess changes in *S*. *mansoni* miracidia behaviour, including: i) movement towards the zone of application; ii) restricted movement within the application zone; and iii) significantly increased velocity, angular variation and tortuosity movements.

**Fig 3:**
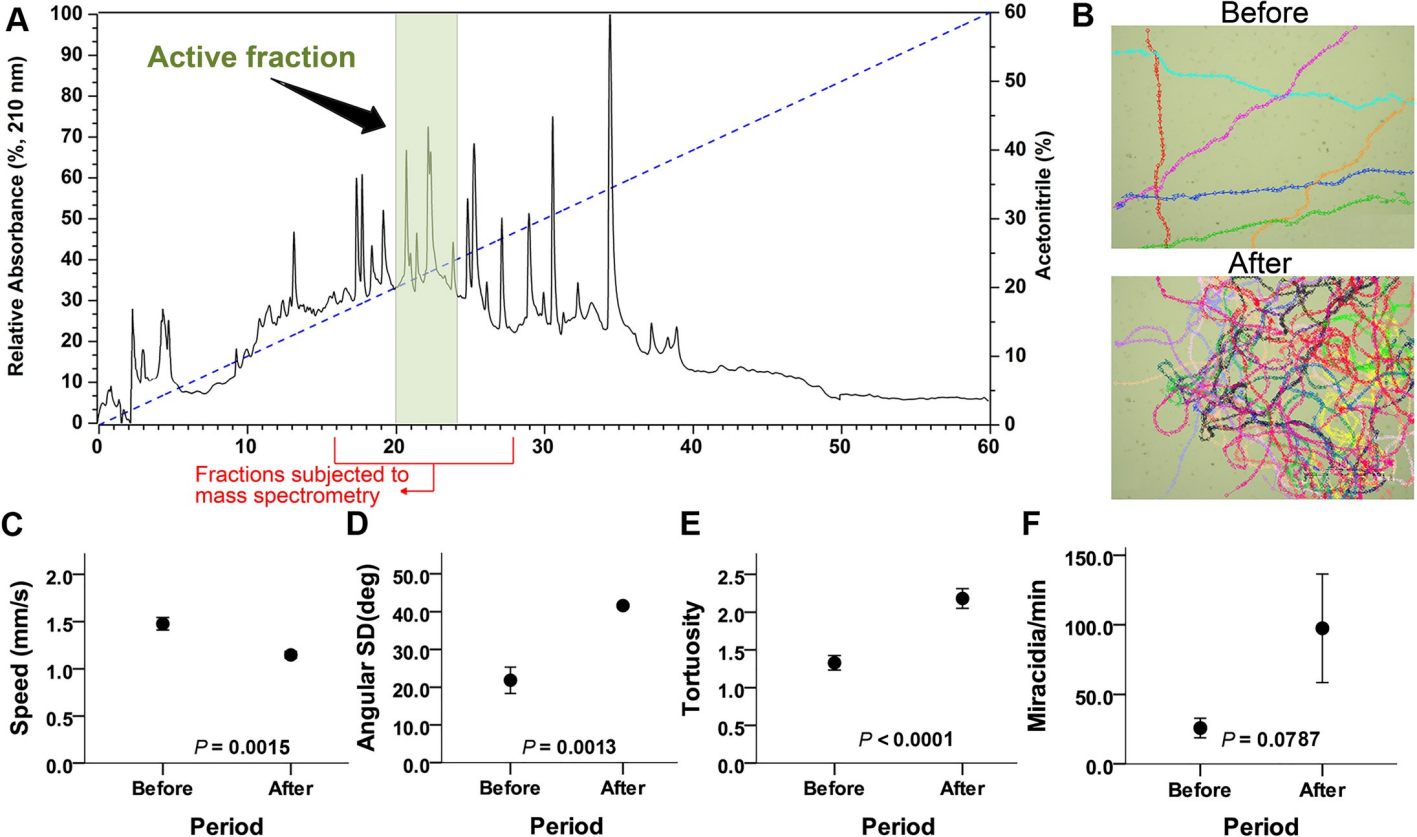
Identification of bioactive RP-HPLC fraction from *B*. *glabrata* conditioned water. (**A**) Representative RP-HPLC chromatogram of SCW crude extracts of *B*. *glabrata*, with the active fraction highlighted. (**B**) Representative movement trajectories and density of miracidia 1 min before and 1 min after the addition of 2 μl of active fraction as a resuspended solution (protein concentration ~1 mg/ml). Each colour represents the swim-path of one tracked miracidium. See **S5 Movie** for the assay video. (**C-F**) Analyses of active fraction effect on miracidia behaviour measurements (means ± standard error). For each measurement type, paired t-tests (n = 6 for each) were used to compare measurements before and after addition of the bioactive HPLC fraction. Miracidia speed (**C**), angular standard deviation (**D**), track tortuosity (**E**) and rate of accumulation (**F**) before and after RP-HPLC fraction 20–25 min application.

A single HPLC fraction (20–25 min), highlighted in **Fig 3A**, demonstrated behaviour modification bioactivity in assays, as shown in **Fig 3B** and **S5 Movie**. The miracidium trajectories, swim time within the application zone and mean acceleration magnitudes were compared. A significant increase in miracidia density was observed within the application zone, while miracidia distribution outside this zone diminished, indicating that this fraction contained a biomolecule that triggered a rapid response in the miracidia. Miracidia velocity was reduced (**Fig 3C**), turning (as measured by the variation in heading) was increased (**Fig 3D**), resulting in more tortuous paths (**Fig 3E**). Also, miracidia rate of accumulation increased after RP-HPLC fraction 20–25 min application (**Fig 3F**). All three individual measures of behaviours (speed, angular standard deviation, and tortuosity) showed significant changes after adding the fraction. The group measure of the aggregation rate of miracidia was also substantially higher after fraction addition, but very high variability meant the difference was marginally above the two-tailed threshold for significance.

### The analysis of *Biomphalaria*-derived biomolecules in the HPLC fraction

Metabolites and proteins present within the active HPLC fraction were identified using UHPLC-QTOF and nanoHPLC tandem triple-TOF electrospray mass spectrometry, respectively; two adjacent fractions were likewise analysed. In total, we found ~16 metabolites, all of which did not match to any known compound within the METLIN [27] or HMDB [28] metabolite databases (**S1 Table**). Also in the active HPLC fraction, a total of 24 peptides were identified that could be matched to precursor proteins in the genome-derived *B*. *glabrata* protein database [42]. Several peptides identified within the fractions (false discovery rate <1%) are cleaved from 4 different *B*. *glabrata* precursor proteins (**Table 1**) with MS/MS spectra displayed in **S1 File**, each of which contain a signal peptide indicative of processing through a classical secretion pathway. BLASTp analysis indicated that they had no match with any known protein present within NCBI databases (E-value cut-off 10^−5^).

**Table 1:** Proteins identified from the active fraction by LC-MS/MS (see S1 File for more details).

Protein Group	Accession	-10lgP	Coverage (%)	#Peptides	Mass (Dalton)	Signal peptide cleavage pos. (residue number)
1	Locus_84615_Transcript_1/1	336.49	79	23	9975	N/A
2	Locus_39923_Transcript_1/2	127.48	18	5	22840	20/21
3	Locus_12313_Transcript_1/2	102.47	15	4	20112	18/19
3	Locus_12313_Transcript_2/2	102.47	15	4	20142	18/19
4	Locus_32929_Transcript_1/2	34.5	5	1	20714	18/19
4	Locus_17865_Transcript_7/8	34.5	5	1	21105	18/19

### Screen for activity of identified peptides

**Fig 4:**
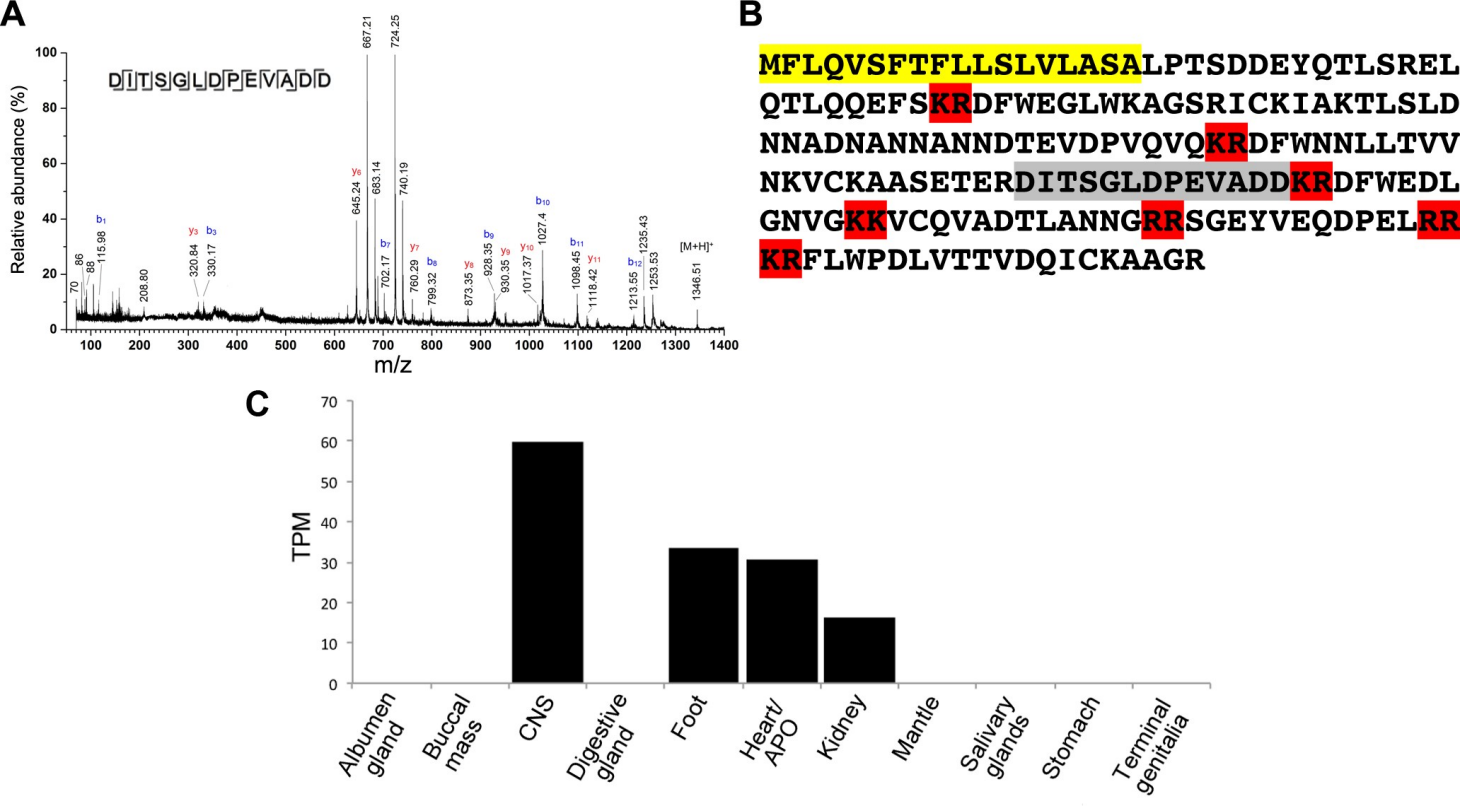
Identification of *B*. *glabrata* P12 peptide and tissue expression. (**A**) MS/MS spectrum of peptide P12—DITSGLDPEVADD-*OH*. (**B**) P12 precursor protein showing signal peptide (yellow), potential cleavage sites (red) and region of P12 (grey). (**C**) P12 gene expression in various *B*. *glabrata* tissues. TPM, transcripts per kilobase million mapped reads as determined by *de novo* RNA-seq [42] (CLC Genomic Workbench 9 software). APO, amebocyte-producing organ.

All peptides were synthesised (**S2 Table**) and tested in bioassay on *S*. *mansoni* miracidia. Most peptide fractions produced no substantial responses from miracidia (combined analysis in **S2 Fig**), however peptide “P12” demonstrated bioactivity. P12 consists of a 13-residue peptide (DITSGLDPEVADD) based on mass spectrometry analysis of the HPLC-bioactive fraction (**Fig 4A**). The P12 precursor protein is 186 amino acids in length, containing an 18-residue signal peptide and multiple dibasic sites that are likely cleaved (**Fig 4B**). Gene transcripts that encode the P12 were identified with relatively high expression in the *B*. *glabrata* central nervous system, and are also present in the foot, heart/amebocyte-producing organ and kidney (**Fig 4C**). No gene expression was observed in other tissues.

**Fig 5:**
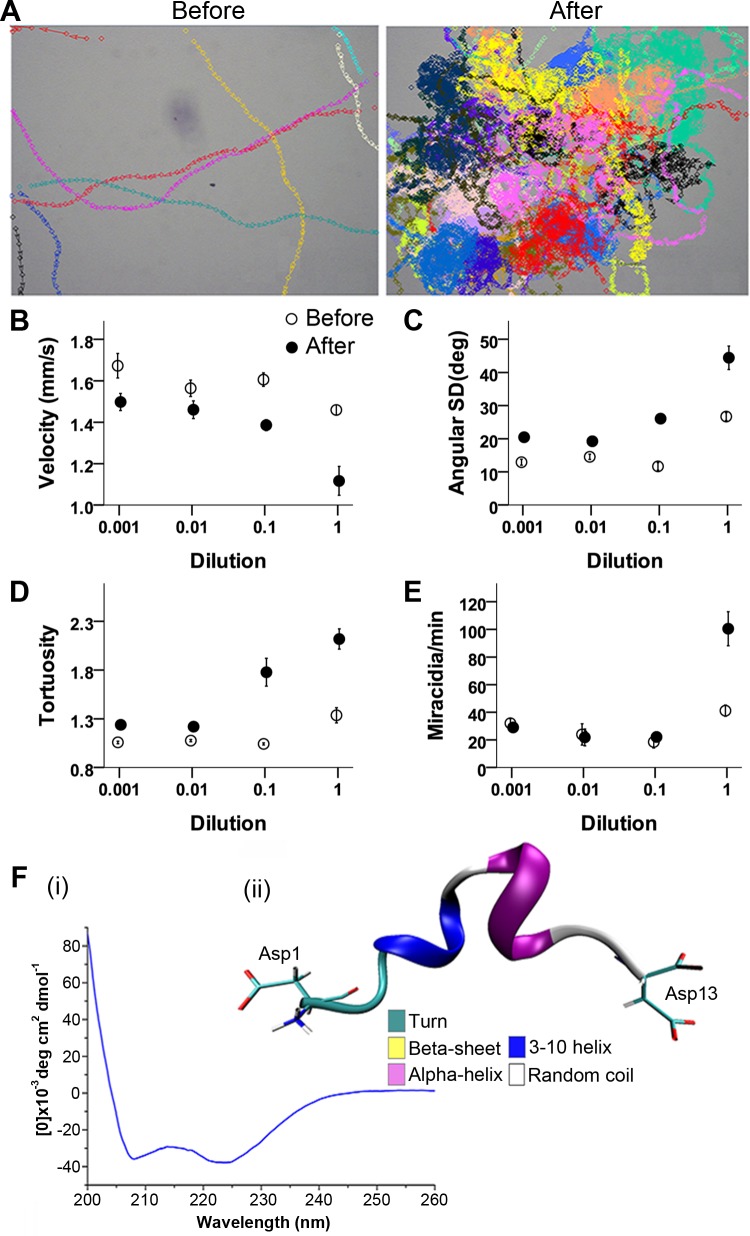
Bioassay and structure analysis of *B*. *glabrata* P12 peptide. (**A**) Representative trajectories of miracidia movement before and after the addition of 2 μl of the P12 solution (0.742 μM) to the centre of the area. Each colour represents one indistinguishable miracidium individual. See **S6 Movie** for assay video. Miracidia speed (**B**), angular standard deviation (**C**), track tortuosity (**D**) and rate of accumulation (**E**) before and after P12 application at four different dilutions (means ± standard errors). (**F**) Structural analysis of P12. (i) Circular dichroism spectrum of P12, and (ii) representative structure of P12 at 306.4 K during the REMD simulation.

P12 peptide bioactivity was demonstrated by induction of changes in RCD (**S6 Movie**), similar to that described for raw mucus extracts and the bioactive HPLC fraction. Example trajectories of miracidia movement before and after addition of P12 (2 μl at 7.42 μM) are shown in **Fig 5A**. When tested across multiple trials (n = 6 per dilution level), P12 caused clear changes in miracidia behaviour that depended on concentration: velocity was reduced (**Fig 5B**), turning (as measured by the variation in heading) was increased (**Fig 5C**), resulting in more tortuous paths (**Fig 5D and Table 2**). A similar change in behaviour was also observed in the bioactive HPLC fraction (see **Fig 3C**). The consequence of these P12-induced changes is that the miracidia transition from swimming in relatively straight paths to highly convoluted paths close to where the peptide was applied. Miracidia thus congregate at significantly greater rates at the site of application. Serial dilution assays (including 10×, 100×, and 1000× dilutions) of P12 consistently showed diminishing but still significant effects on swimming velocity, turning and tortuosity (**S7**and **S8 Movies**). The consequence of these reduced effects on individual behaviours was to eliminate the group congregation effect at lower concentrations of the peptide (**Fig 5E and S3 Table**). Following these acute effects, miracidia behaviour was observed at 30 min post-application, showing obvious termination in movement, replaced by either extension and contraction movements, or small magnitude rotations (**S9**and **S10 Movies**); this behaviour had also been observed 30 min post-application of raw mucus, suggesting P12, if not the primary inducer, is capable of eliciting this physiological response by itself.

**Table 2:** Repeated measures MANOVA tests of differences between miracidia behavioural measurements before and after application of peptide P12. Significant before vs after within-subjects tests indicate that regardless of dilution, P12 significantly affected the behavioural measurements, while concomitant significant interaction tests indicate that the magnitude of the P12 effect depended on the dilution level, as would be expected for a bioactive molecule. Standard deviation, SD; degrees of freedom, df.

Measurement	Test	F	df	P-value
Speed	Before vs After	127.2	1,20	<0.0001
	Dilution	22.3	3,20	<0.0001
	Interaction	7.2	3,20	0.0018
Angular SD	Before vs After	198.5	1,20	<0.0001
	Dilution	39.7	3,20	<0.0001
	Interaction	16.2	3,20	<0.0001
Tortuosity	Before vs After	115.6	1,20	<0.0001
	Dilution	22.3	3,20	<0.0001
	Interaction	16.2	3,20	<0.0001
Miracidia/min	Before vs After	22.5	1,20	0.0001
	Dilution	18.6	3,20	<0.0001
	Interaction	24.4	3,20	<0.0001

The circular dichroism (CD) spectrum of the P12 peptide was investigated, indicating a helix structure in low concentration saline buffer (**Fig 5F**). In addition, the structure of P12 in water was theoretically predicted using replica exchange molecular dynamic simulation, which has been reported as a viable method in structural prediction and analysis for peptides [43–47]. The conformations at 306.5K were extracted, and their secondary structures evaluated through the simulation (**S2 Fig**). P12 displays a degree of α-helix and/or 3–10 helix secondary structural conformation, which are mainly distributed in the regions Ile3-Ser4 and Pro8-Val10 (**Fig 5F**). This replica-exchange molecular dynamics simulation is in good agreement with CD spectroscopy. There are a number of conformations showing high content of ‘turn’ (hydrogen bonded turn) at these two regions, especially at Asp7-Ala11, also an indication of the stability. A random coil occurs at N-/C- terminus and Leu6. This ‘helix-hinge-helix’-like structure lends further support to the proposed stability of P12 in water [48, 49].

## Discussion

The primary goal of this study was to develop an approach for elucidating bioactive component(s) (kairomones) released from an intermediate aquatic parasite host, which facilitates parasite host identification. Previous studies have only reported on non-specific small molecular weight biomolecules [21] or MAGs present within SCW, which had been implicated following observed changes to miracidial RCD and turn-back responses [18, 24]. Our current study reports on the purification, bioassay and characterisation of a *B*. *glabrata*-derived peptide that clearly induces behaviour changes in the *S*. *mansoni* miracidia.

First, our study confirmed that raw mucus from *B*. *glabrata* (strain BB02) could stimulate behaviour changes in *S*. *mansoni* (Puerto Rican strain) miracidia, as determined by an increase in total acceleration/de-acceleration movements. In addition, velocity and angular variation behavioural patterns became apparent, in alignment with previously classified miracidial responses [19, 50–52]. The localised movement, followed by repeated extension and contraction, as well as periods of rapid rotation from their anterior point, have been described in transformation assays of miracidia under different culture conditions [53–56]. These are subsequently followed by miracidial ciliated plate shedding and development into the successive sporocyst life-stage. We similarly showed that snail crude mucus and SCW that had been filtered (0.45 μm filter) retained the bioactive properties. In summary, our bioassay-guided analysis of crude mucus and SCW extracts, as well as semi-purified extracts, confirmed that *B*. *glabrata* release a molecule(s) that can modify the swimming behaviour of *S*. *mansoni* miracidia, which likely enables species-sensitive chemosensory detection.

The identity of *Biomphalaria*-derived biomolecule(s) that stimulated behaviour changes in *S*. *mansoni* miracidia, required the proteomic analysis of the filtered SCW. Based on our previous experiments, we hypothesised that, prior to the addition of an active fraction, *S*. *mansoni* miracidia behaviour would exhibit patterns of random movement, with statistically equal distribution throughout the Petri dish, at a stable velocity, whereas following the addition of a bioactive fraction, miracidia movement would be altered. This was most prominent in the single HPLC fraction at 20–25 min (see **S5 Movie**), including significant changes in speed, angular standard deviation, and tortuosity. The active HPLC fraction contained a total of 24 peptides, of which several peptides were cleaved from 4 different *B*. *glabrata* precursor proteins that appeared to be unique to *B*. *glabrata*, consistent with the requirement of species-specificity. However, *Biomphalaria* is currently the only planorbid species (air-breathing freshwater snails) with extensive gene data available.

The P12 peptide consists of 13 residues (DITSGLDPEVADD) that is derived from a precursor protein of 186 amino acids. Although the Neuropred tool does not confidently predict precursor cleavage at the N-terminal arginine flanking the P12 peptide, other studies have reported cleavage may occur at single arginine residues (e.g. in human blood coagulation factor IX [57]). The observation of relatively high gene expression in the central nervous system, foot, heart and kidney of *B*. *glabrata*, based on RNA-seq, could support the fact that snails have an open circulatory system and hemolymph components can be actively transported externally. P12 peptide bioactivity was demonstrated by induction of changes in RCD (see **S6 Movie**), and further structural analysis demonstrating a helix-hinge-helix structure suggests stability in water. Thus, upon approaching a cell membrane (as may occur when encountering a cell surface receptor), a stable native structure could be expected, which might favour the interaction between P12 and its potential receptor.

In conclusion, in this study we have reported the identification of a peptide, P12, which is secreted from adult *B*. *glabrata* and triggers extreme behaviour modifications in the *S*. *mansoni* miracidia. The P12 peptide, and its precursor appear to be unique to this snail, thus providing an ideal species-specific chemosensory cue for *S*. *mansoni* miracidia. This finding contributes greatly to our understanding of a key part within the parasite’s life-cycle, and may help towards establishing novel biocontrol interventions for schistosomiasis. We propose, for example, that an artificial concentrated ‘P12-cloud’ could be created that may attract *S*. *mansoni* miracidia away from *Biomphalaria*, thus preventing snail infection. Our knowledge of the peptide structure may also be useful towards designing agonists, as well as defining the receptor to which it binds. In addition, the workflow we report could help to identify similar parasite-host kairomones, used in other species.

## Supporting information

S1 Fig Behaviour of miracidia before and after the addition of SCW of *Oncomelania quadrasi* and *Theba pisana*. Before addition, and after addition of SCW, showing acceleration magnitude. See **S3**and **S4 Movies** for assay videos.
(DOCX)

S2 Fig Grouped analyses of all other synthetic peptides (excluding P12) on miracidia behaviour measurements. For each measurement type, paired t-tests (n = 12 for each) were used to compare measurements before and after addition of these apparently inactive peptides.
(DOCX)

S3 Fig Secondary structure information of the 100,000 conformations of P12 at 306.4K obtained by REMD simulation. (DOCX)

S1 Table Empirical formula generated from three different HPLC fractions of *B*. *glabarata*. The proposed formula obtained after the multivariate data analysis according to high-resolution LC-QToF-MS measurements.
(DOCX)

S2 Table Synthetic peptides for *S*. *mansoni* miracidium behaviour change activity test. (DOCX)

S3 Table Summary statistics and paired t-tests (n = 6 in all cases) comparing miracidia behaviour measurements before and after application of different dilutions of P12. Given that repeated measures MANOVA (Table 2 in the main text) indicated significant interactions between the effect of P12 and dilution, bold P-values indicate a significant difference between before and after P12 application at that dilution. Standard deviation (SD); Standard error of the mean (SEM).
(DOCX)

S1 File Mass spectra and SignalP results for proteins present within bioactive HPLC fraction. (DOCX)

S1 Movie Behaviour of miracidia after the addition of raw mucus of *B*. *glabrata* (speed 4x). Followed the SCW collection in Methods, mucus on the beaker wall and bottom was collection and subjected to the bioassay directly.
(AVI)

S2 Movie Behaviour of miracidia after the addition of filtered *B*. *glabrata* SCW (Speed 2x). The SCW after filtration using 0.45 μm PVDF Millex-HV was lyophilised, and 1 mg of extracts was resuspended in pH neutral water and tested.
(AVI)

S3 Movie Behaviour of miracidia after the addition of crude extracts of *Oncomelania quadrasi* SCW (Speed 4x). The preparation method was similar to *B*. *glabrata*.
(AVI)

S4 Movie Behaviour of miracidia after the addition of crude extracts of *Theba pisana* SCW (Speed 4x). The preparation method was similar to *B*. *glabrata*.
(AVI)

S5 Movie Behaviour of miracidia after the addition of the active fraction purified by HPLC (see Methods) from SCW of *B*. *glabrata* (Speed 2x). Videos of other fractions without activity are not shown.
(AVI)

S6 Movie Behaviour of miracidia after the addition of peptide P12 (7.42 μM) (Speed 4x). (AVI)

S7 Movie Behaviour of miracidia after the addition of peptide P12 (0.742 μM) (Speed 4x). (AVI)

S8 Movie Behaviour of miracidia after the addition of peptide P12 (74.2 nM) (Speed 4x). (AVI)

S9 Movie Behaviour of miracidia 30 min post-addition of peptide P12 (7.42 μM) (Speed 2x). (AVI)

S10 Movie Behaviour of miracidia 30 min post-addition of peptide P12 (74.2 nM) (Speed 2x). (AVI)
