# Supplementary material for: A Biomphalaria glabrata peptide that stimulates significant behaviour modifications in aquatic free-living Schistosoma mansoni miracidia
Source: PLoS Negl Trop Dis. 2019 Jan 22;13(1):e0006948. doi: 10.1371/journal.pntd.0006948 (PMC6358113; doi:10.1371/journal.pntd.0006948)
Supplement: S1 Fig — Before addition, and after addition of SCW, showing acceleration magnitude. See S3and S4 Movies for assay videos. (DOCX) [file pntd.0006948.s001.docx]

**
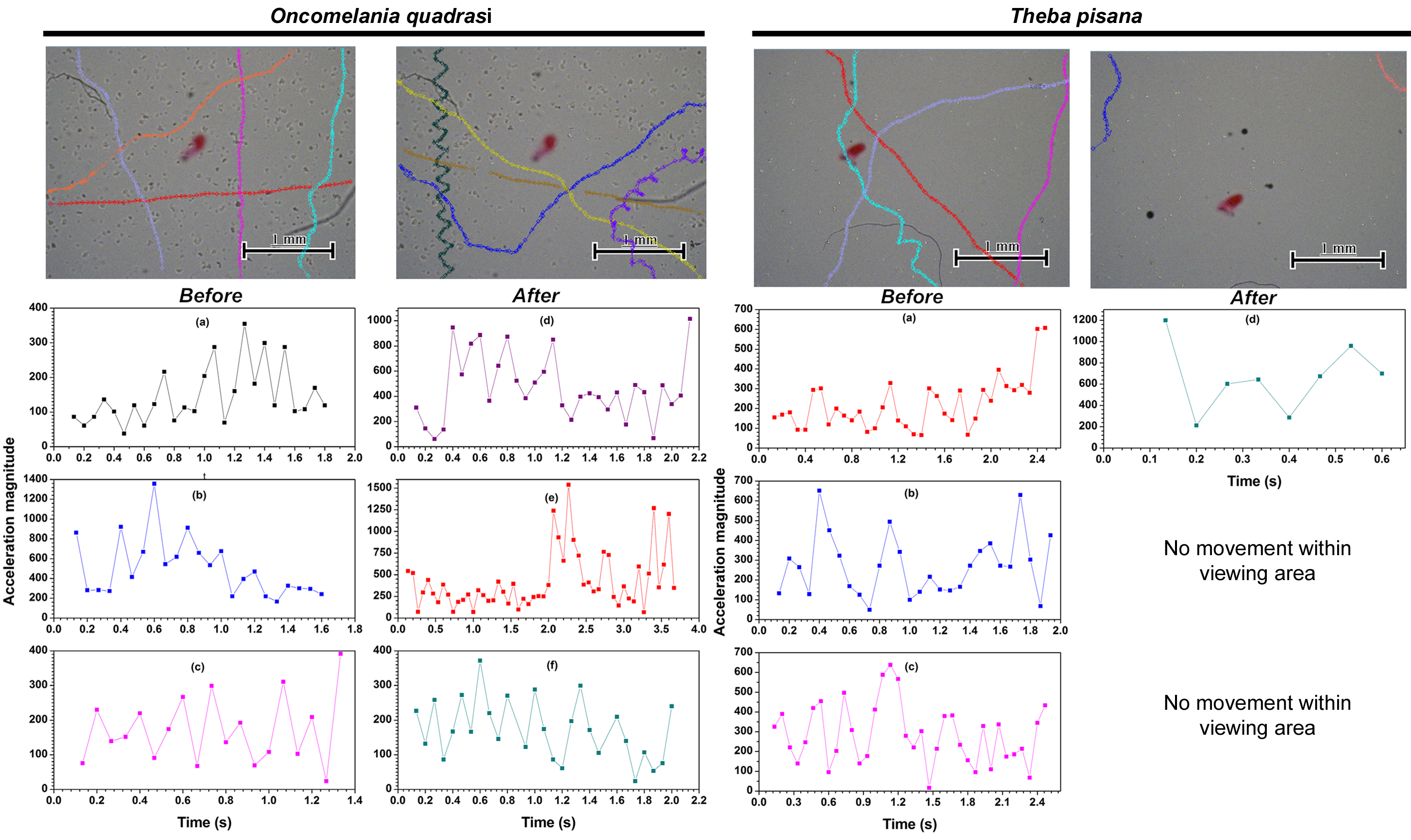
**

**S1 Figure.** Behaviour of miracidia before and after the addition of SCW of *Oncomelania quadrasi* and *Theba pisana*. Before addition, and after addition of SCW, showing acceleration magnitude. See **Movie S3** and **Movie S4** for assay videos.
