# Supplementary material for: A Biomphalaria glabrata peptide that stimulates significant behaviour modifications in aquatic free-living Schistosoma mansoni miracidia
Source: PLoS Negl Trop Dis. 2019 Jan 22;13(1):e0006948. doi: 10.1371/journal.pntd.0006948 (PMC6358113; doi:10.1371/journal.pntd.0006948)
Supplement: S2 Fig — For each measurement type, paired t-tests (n = 12 for each) were used to compare measurements before and after addition of these apparently inactive peptides. (DOCX) [file pntd.0006948.s002.docx]

**
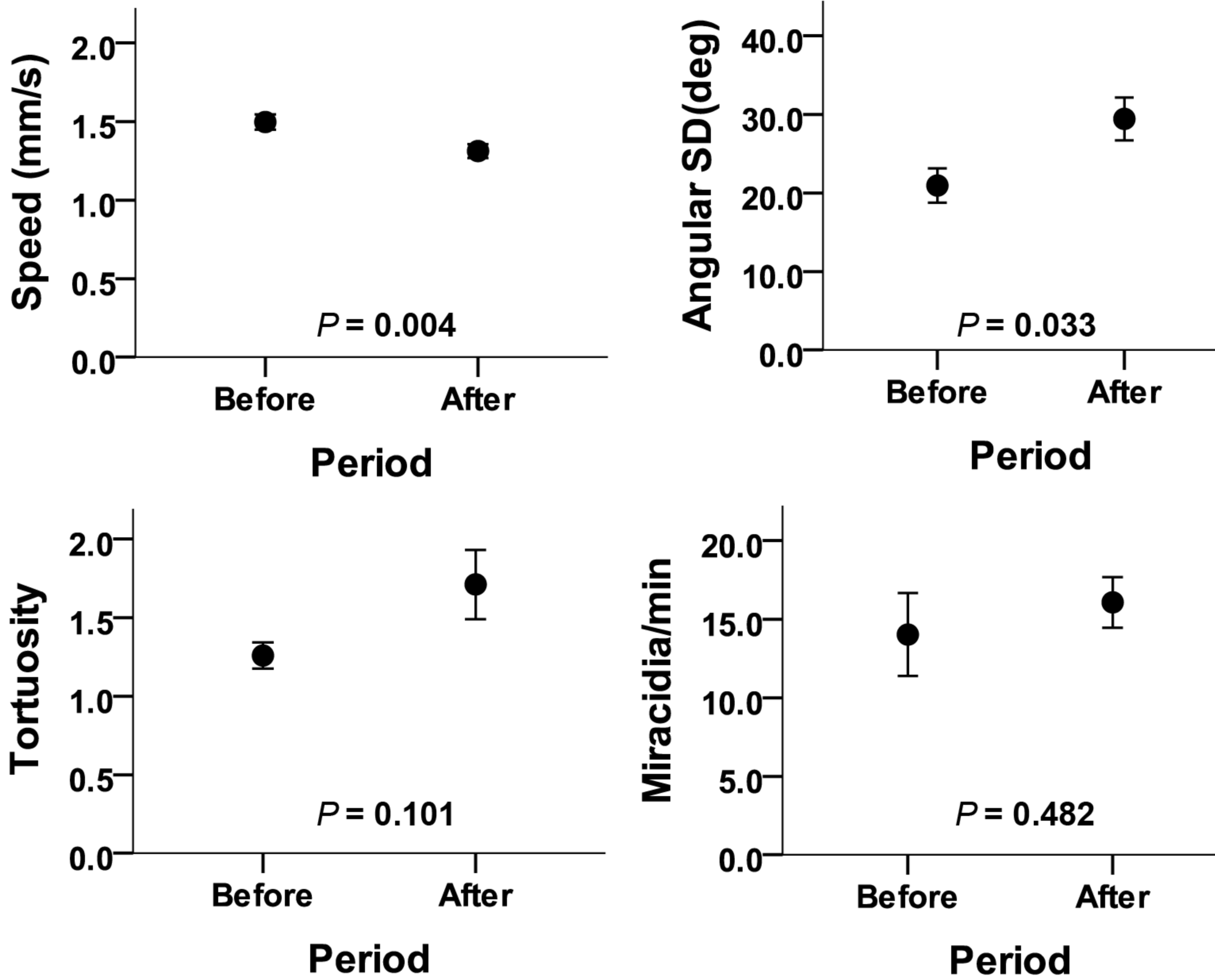
**

**S2 Figure.** Analyses of synthetic peptides (minus P12) on miracidia behaviour measurements. For each measurement type, paired t-tests (n = 12 for each) were used to compare measurements before and after addition of these apparently inactive peptides.
