# Supplementary material for: A Biomphalaria glabrata peptide that stimulates significant behaviour modifications in aquatic free-living Schistosoma mansoni miracidia
Source: PLoS Negl Trop Dis. 2019 Jan 22;13(1):e0006948. doi: 10.1371/journal.pntd.0006948 (PMC6358113; doi:10.1371/journal.pntd.0006948)
Supplement: S3 Fig — (DOCX) [file pntd.0006948.s003.docx]

**
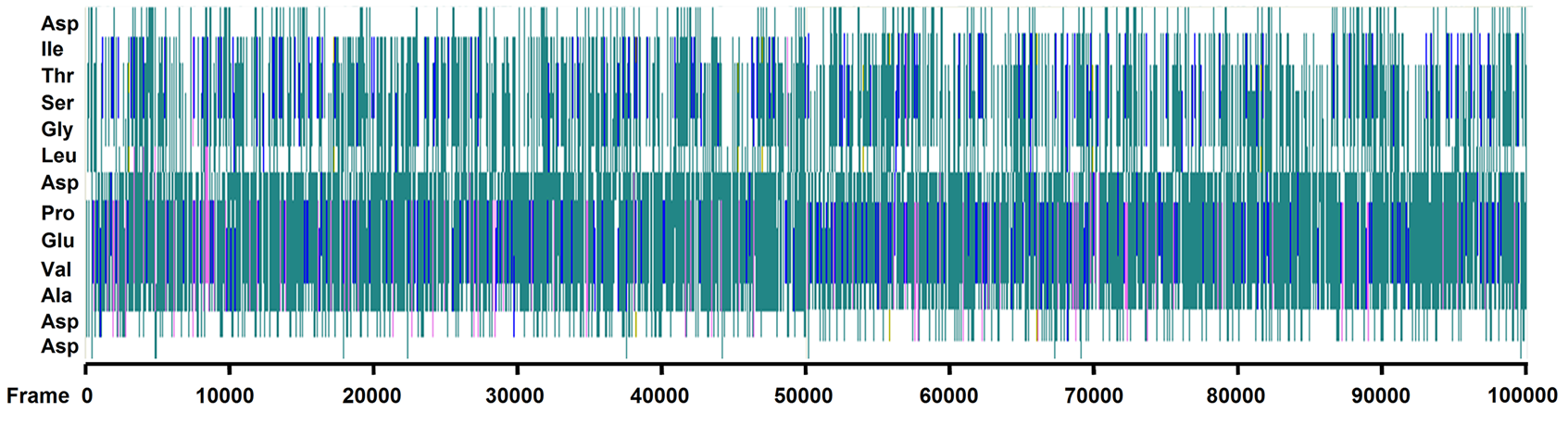
**

**S3 Figure.** Secondary structure information of the 100,000 conformations of P12 at 306.4 K obtained by REMD simulation. Colour representations: purple- α helix, blue- 3-10 helix, cyan- turn and white- random coil.
