# Supplementary material for: A Biomphalaria glabrata peptide that stimulates significant behaviour modifications in aquatic free-living Schistosoma mansoni miracidia
Source: PLoS Negl Trop Dis. 2019 Jan 22;13(1):e0006948. doi: 10.1371/journal.pntd.0006948 (PMC6358113; doi:10.1371/journal.pntd.0006948)
Supplement: S1 Table — The proposed formula obtained after the multivariate data analysis according to high-resolution LC-QToF-MS measurements. (DOCX) [file pntd.0006948.s004.docx]

**S1 Table.** Empirical formula generated from three different HPLC fractions of *B. glabarata*. The proposed formula obtained after the multivariate data analysis according to high-resolution LC-QToF-MS measurements.

| Molecular Formula | Overall  Score^*^ | Mass^**^ | Polarity | RT (min) | Difference  (MFG, ppm) |
| --- | --- | --- | --- | --- | --- |
| C_4_H_5_N_3_O | 98.35 | 112.0509 | Positive | 6.82 | -3.04 |
| C_5_H_5_N_5_O | 82.84 | 152.0573 | Positive | 6.89 | -2.88 |
| C_24_H_34_N_2_O_­_ | 81.01 | 367.2751 | Positive | 13.65 | 2.46 |
| C_13_H_25_NO_3_ | 77.19 | 226.1812 | Positive | 19.99 | 2.75 |
| C_19_H_33_NO | 78.02 | 292.2648 | Positive | 20.92 | 3.89 |
| C_25_H_26_N_4_O_2_ | 99.09 | 415.2145 | Positive | 28.29 | 0.89 |
| C_19_H_41_N | 81.92 | 284.3323 | Positive | 28.98 | 3.95 |
| C_21_H_45_N | 81.55 | 312.3637 | Positive | 31.06 | 3.59 |
| C_22_H_47_N | 77.95 | 326.3791 | Positive | 31.52 | -2.51 |
| C_15_H_23_N_5_O_4_ | 82.44 | 322.1885 | Positive | 6.63 | -3.3 |
| C_17_H_37_N | 81.65 | 256.3010 | Positive | 26.35 | -3.57 |
| C_8_H_10_N_4_O_3_ | 76.63 | 209.0670 | Negative | 6.92 | 2.9 |
| C_6_H_8_O_7_ | 98.74 | 191.0201 | Negative | 7.42 | -2.3 |
| C_13_H_25_NO_3_ | 78.46 | 242.1768 | Negative | 19.99 | -1.85 |
| C_44_H_37_N_7_O_3_ | 67.64 | 710.7816 | Negative | 27.78 | 0.33 |
| C_15_H_12_N_4_O_7_S | 73.41 | 391.0349 | Negative | 7.39 | 1.07 |

^*^Overall score: For each compound, an overall score is calculated that is based on how well the isotope abundance ratios for the candidate molecular formulas match those from the experimental data. This results in a shorter list of ranked candidate molecular formulas, with the top score (highest score = 100) being more likely to be correct, and therefore increases the value of the accurate-mass analysis.

^**^Mass (*m/z*): the mass to charge ratio of the precursor or largest evidence ion for this compound.
