# Supplementary material for: A Biomphalaria glabrata peptide that stimulates significant behaviour modifications in aquatic free-living Schistosoma mansoni miracidia
Source: PLoS Negl Trop Dis. 2019 Jan 22;13(1):e0006948. doi: 10.1371/journal.pntd.0006948 (PMC6358113; doi:10.1371/journal.pntd.0006948)
Supplement: S2 Table — (DOCX) [file pntd.0006948.s005.docx]

**S2 Table.** Synthetic peptides for *S. mansoni* miracidium-attracting activity test.

| Peptides | Sequence | MW (g mol^-1^) |
| --- | --- | --- |
| P1 | [pGlu]EQAPSQDPSKDADFEQRLS A-OH | 2330.43 |
| P2 | [pGlu]EQAPSQDPSKDADFEQR-OH | 2059.11 |
| P3 | [pGlu]EQAPSQDPSKDADF-OH | 1645.67 |
| P4 | AVQAIKEAAKELESQEGAKES-OH | 2216.45 |
| P5 | IKEAAKELESQEGAKES-OH | 1847.02 |
| P6 | KVAETLEEFKDQPEDAELE-OH | 2220.39 |
| P7 | DEIAKWAPLVF-OH | 1288.52 |
| P8 | [pGlu]EQAPSQDPSKDADFEQRLSAAVQAIKEAAKELESQEGAKES-OH | 4528.86 |
| P9 | VAKVAGAAALVF-NH_2_ | 1115.39 |
| P10 | DFWEDLGNV-NH_2_ | 1093.17 |
| P11 | VCQVA DTLANN-NH_2_ | 1146.29 |
| P12 | DITSGLDPEVADD-OH | 1346.83 |
