# Supplementary material for: A Biomphalaria glabrata peptide that stimulates significant behaviour modifications in aquatic free-living Schistosoma mansoni miracidia
Source: PLoS Negl Trop Dis. 2019 Jan 22;13(1):e0006948. doi: 10.1371/journal.pntd.0006948 (PMC6358113; doi:10.1371/journal.pntd.0006948)
Supplement: S3 Table — Given that repeated measures MANOVA (Table 2 in the main text) indicated significant interactions between the effect of P12 and dilution, bold P-values indicate a significant difference between before and after P12 application at that dilution. Standard deviation (SD); Standard error of the mean (SEM). (DOCX) [file pntd.0006948.s006.docx]

**S3 Table.** Summary statistics and paired t-tests (n = 6 in all cases) comparing miracidia behaviour measurements before and after application of different dilutions of P12. Given that repeated measures MANOVA (Table 2 in the main text) indicated significant interactions between the effect of P12 and dilution, bold P-values indicate a significant difference between before and after P12 application at that dilution. Standard deviation (SD); Standard error of the mean (SEM).

|  |  | Before | | After | | t-test |
| --- | --- | --- | --- | --- | --- | --- |
| P12 Dilution | Measure | Mean | SEM | Mean | SEM | P-value |
| 1X | Speed (mm/s) | 1.46 | 0.02 | 1.12 | 0.07 | **0.004** |
|  | Angular SD (deg) | 26.68 | 1.23 | 44.43 | 3.52 | **0.001** |
|  | Tortuosity | 1.34 | 0.08 | 2.12 | 0.10 | **0.000** |
|  | Miracidia/min | 41.16 | 4.05 | 100.53 | 12.30 | **0.002** |
| 0.1X | Speed (mm/s) | 1.61 | 0.03 | 1.39 | 0.03 | **0.000** |
|  | Angular SD (deg) | 11.64 | 1.17 | 26.04 | 1.31 | **0.000** |
|  | Tortuosity | 1.04 | .01 | 1.78 | 0.14 | **0.003** |
|  | Miracidia/min | 18.46 | 4.29 | 22.11 | 3.78 | 0.545 |
| 0.01X | Speed (mm/s) | 1.56 | 0.04 | 1.46 | 0.04 | **0.005** |
|  | Angular SD (deg) | 14.54 | 0.80 | 19.23 | 1.09 | **0.024** |
|  | Tortuosity | 1.08 | 0.01 | 1.22 | 0.04 | **0.033** |
|  | Miracidia/min | 23.94 | 7.68 | 21.82 | 6.09 | 0.487 |
| 0.001X | Speed (mm/s) | 1.67 | 0.06 | 1.50 | 0.04 | **0.001** |
|  | Angular SD (deg) | 12.93 | 0.91 | 20.47 | 1.40 | **0.000** |
|  | Tortuosity | 1.06 | 0.01 | 1.24 | 0.05 | **0.006** |
|  | Miracidia/min | 32.08 | 3.29 | 28.97 | 2.39 | 0.469 |
