# Supplementary material for: A Biomphalaria glabrata peptide that stimulates significant behaviour modifications in aquatic free-living Schistosoma mansoni miracidia
Source: PLoS Negl Trop Dis. 2019 Jan 22;13(1):e0006948. doi: 10.1371/journal.pntd.0006948 (PMC6358113; doi:10.1371/journal.pntd.0006948)

**S1 File.** Mass spectra and SignalP results for proteins present within bioactive HPLC fraction.

**A. Mass spectra and precursor coverage of peptides (except kairomone P12) identified in bioactive HPLC fractions**


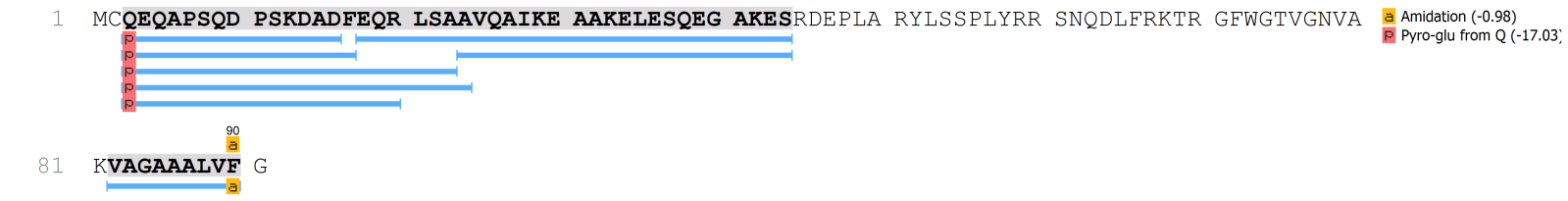


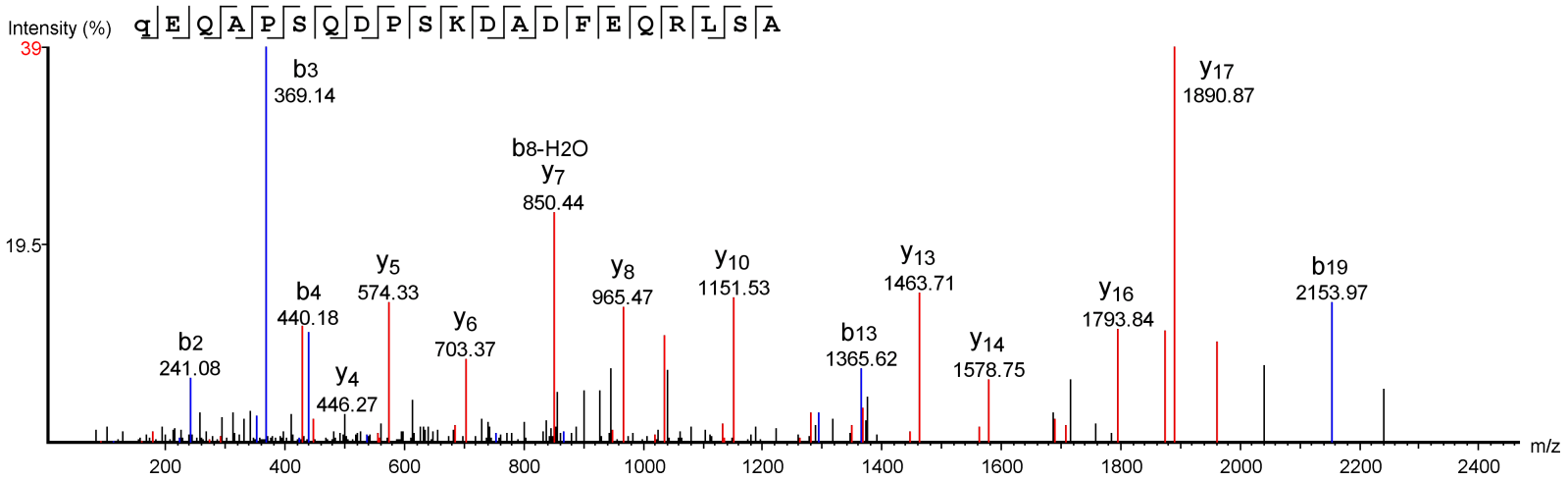


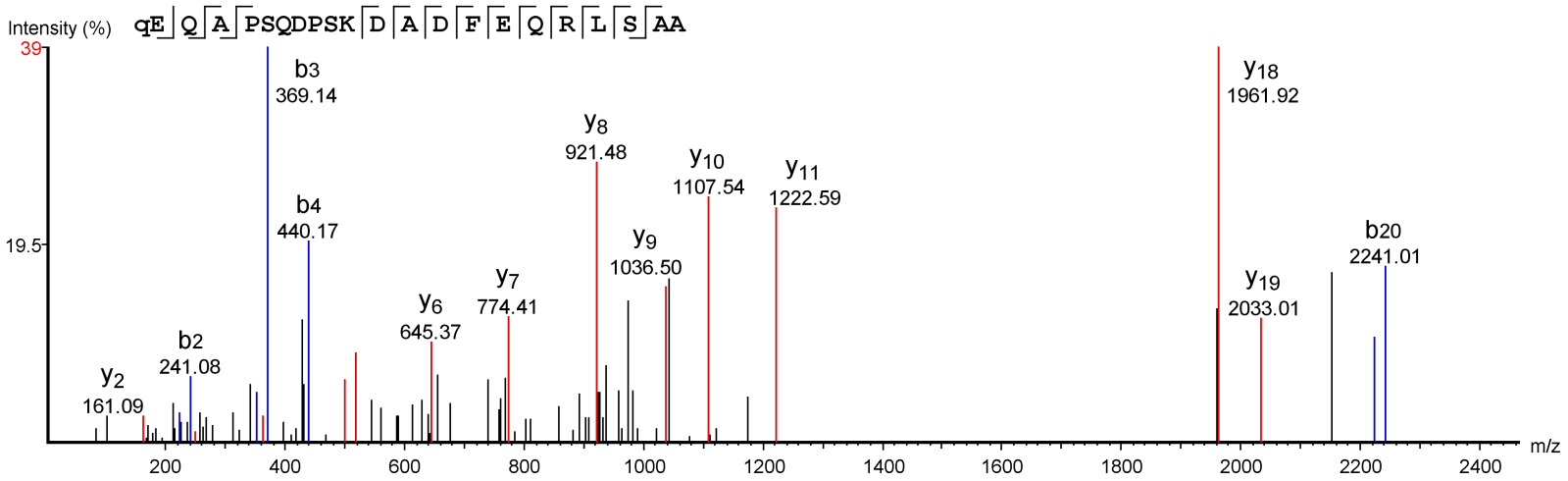


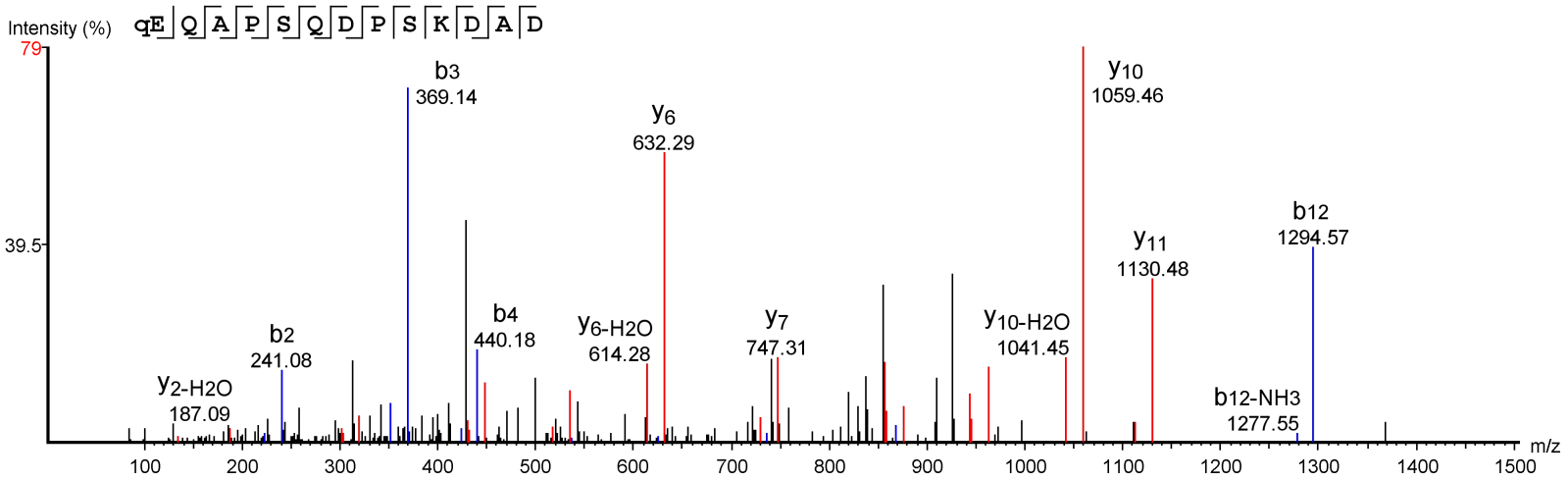


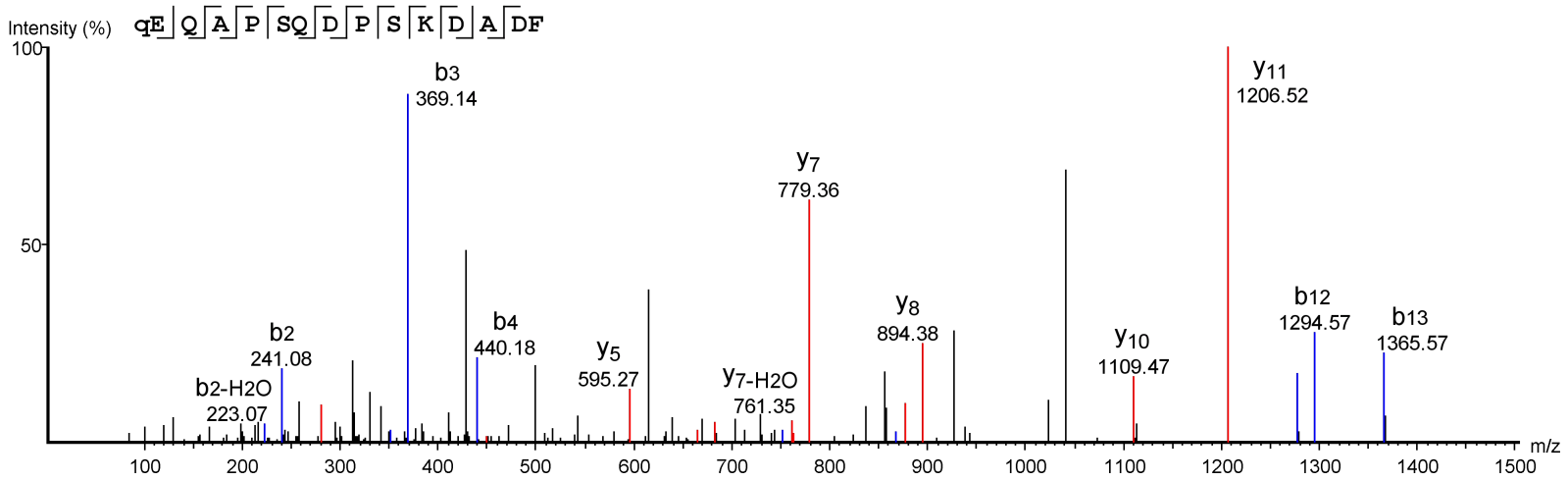


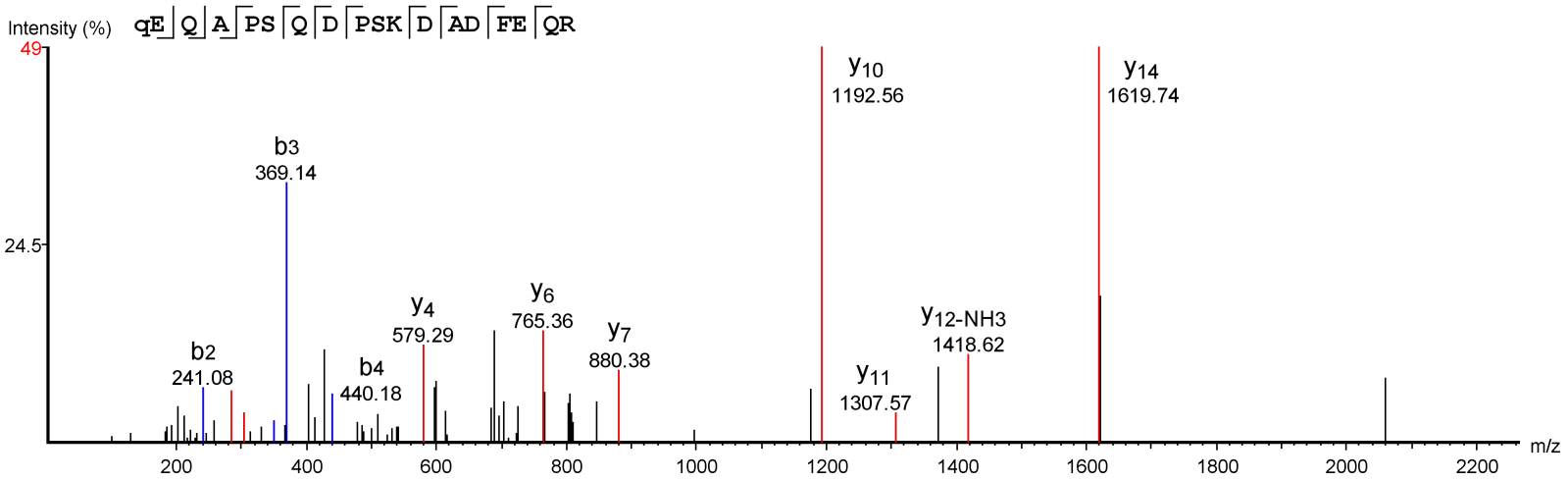


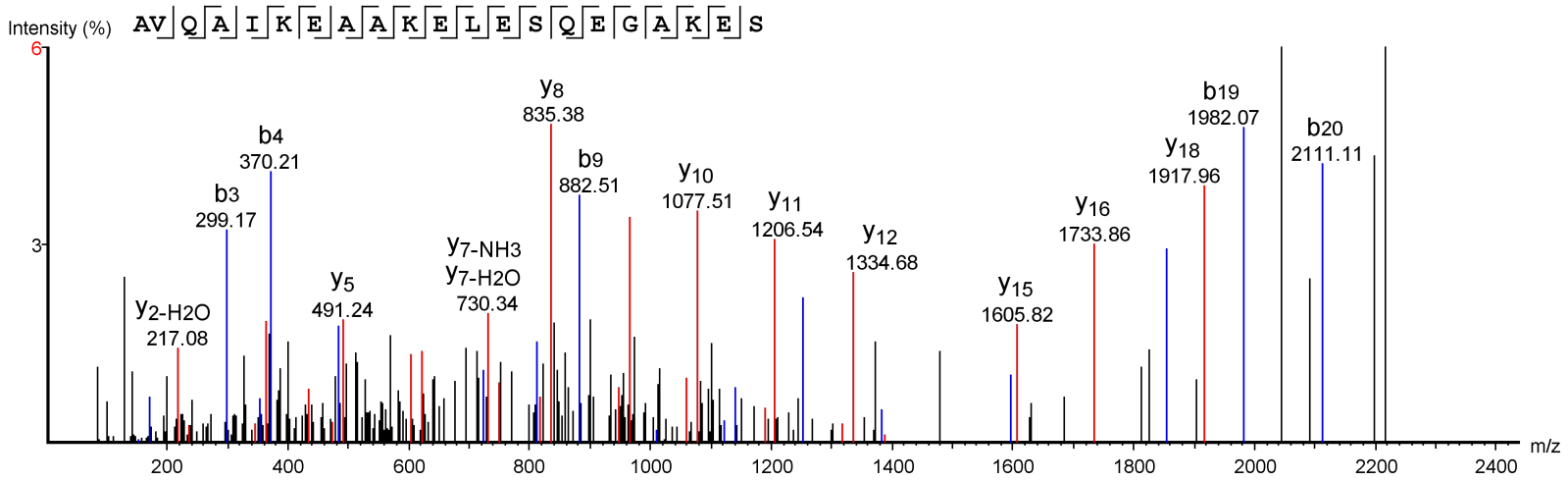


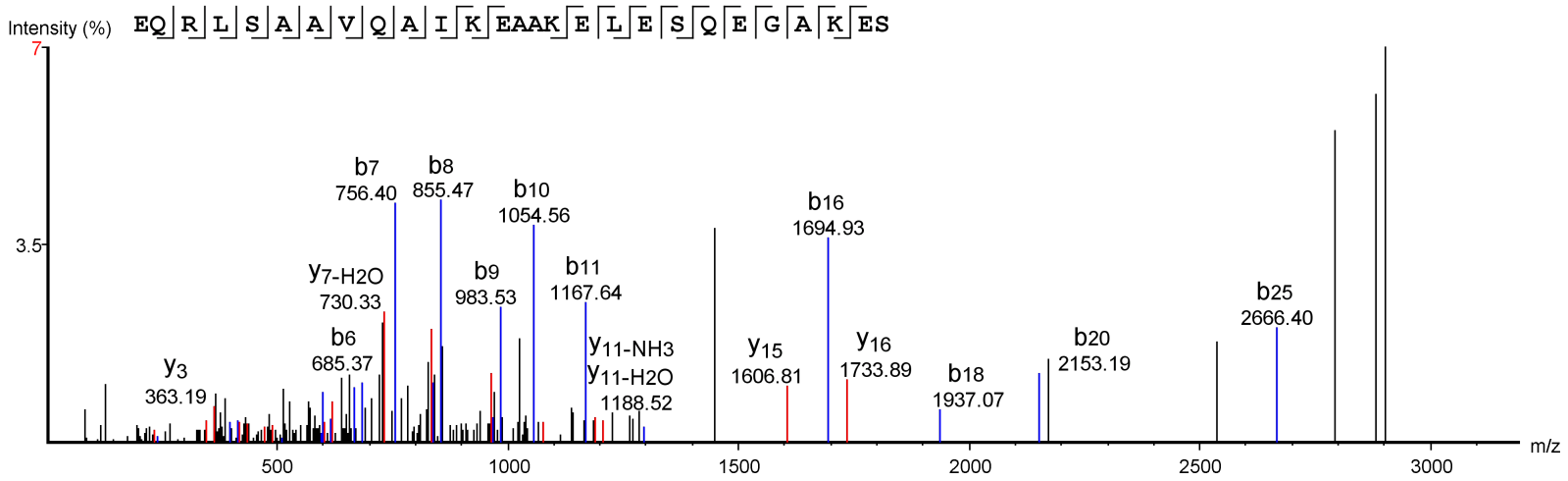


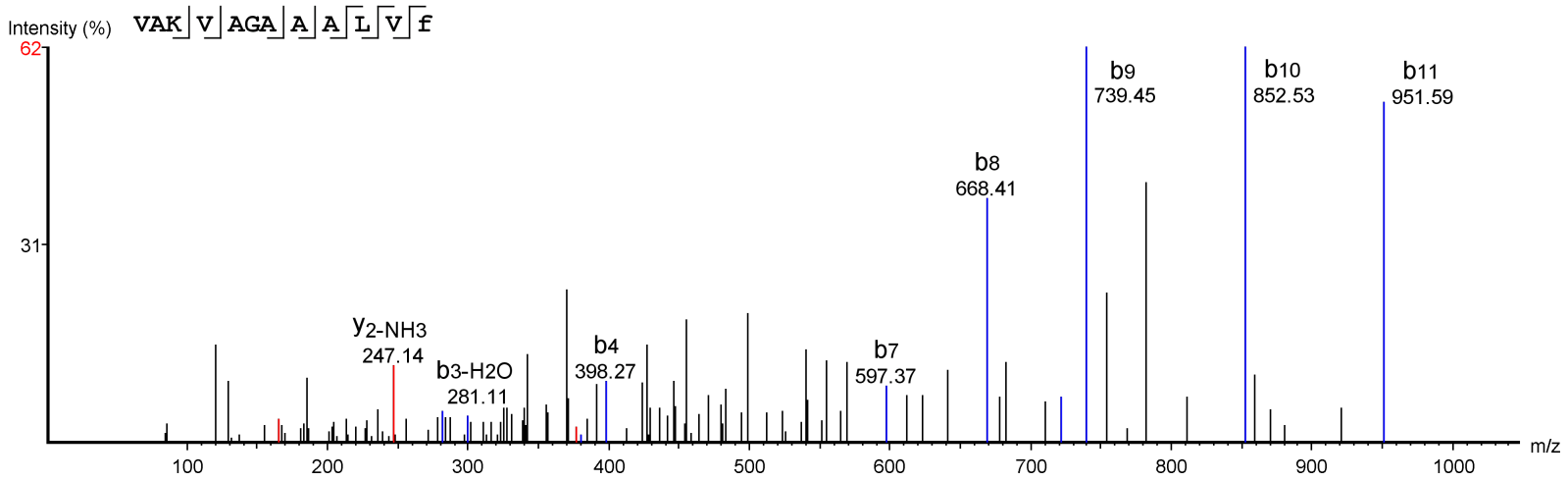


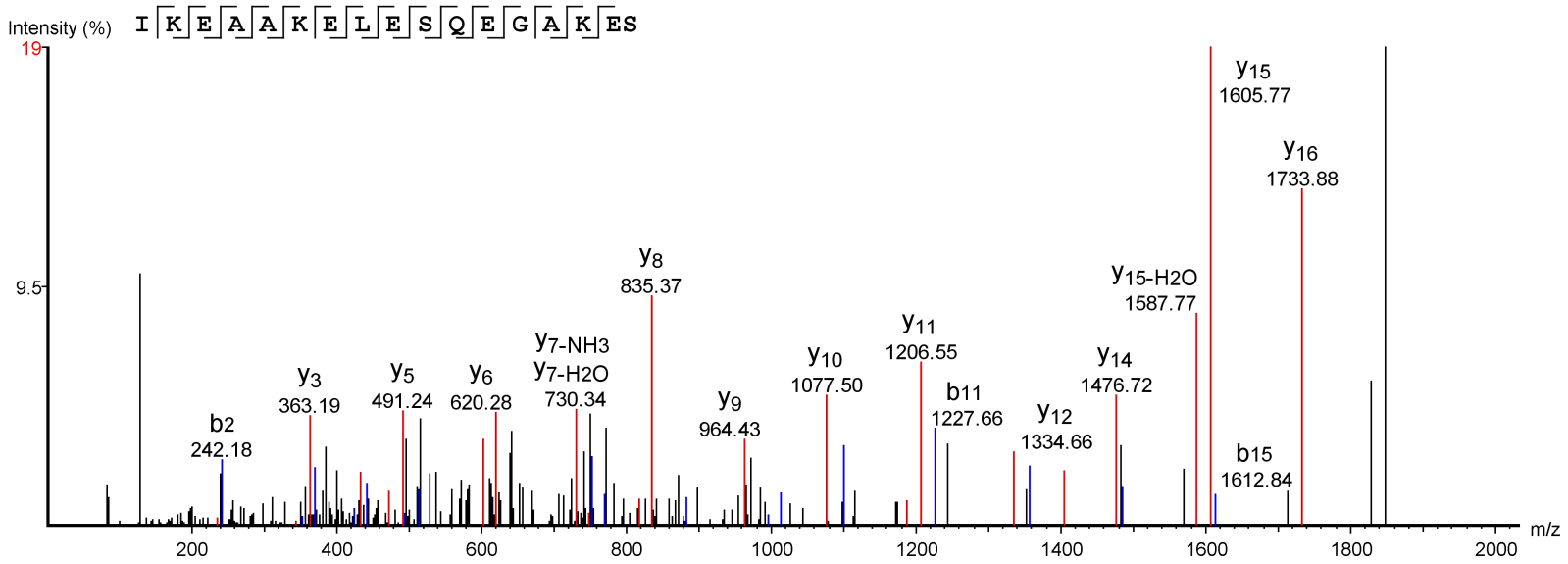


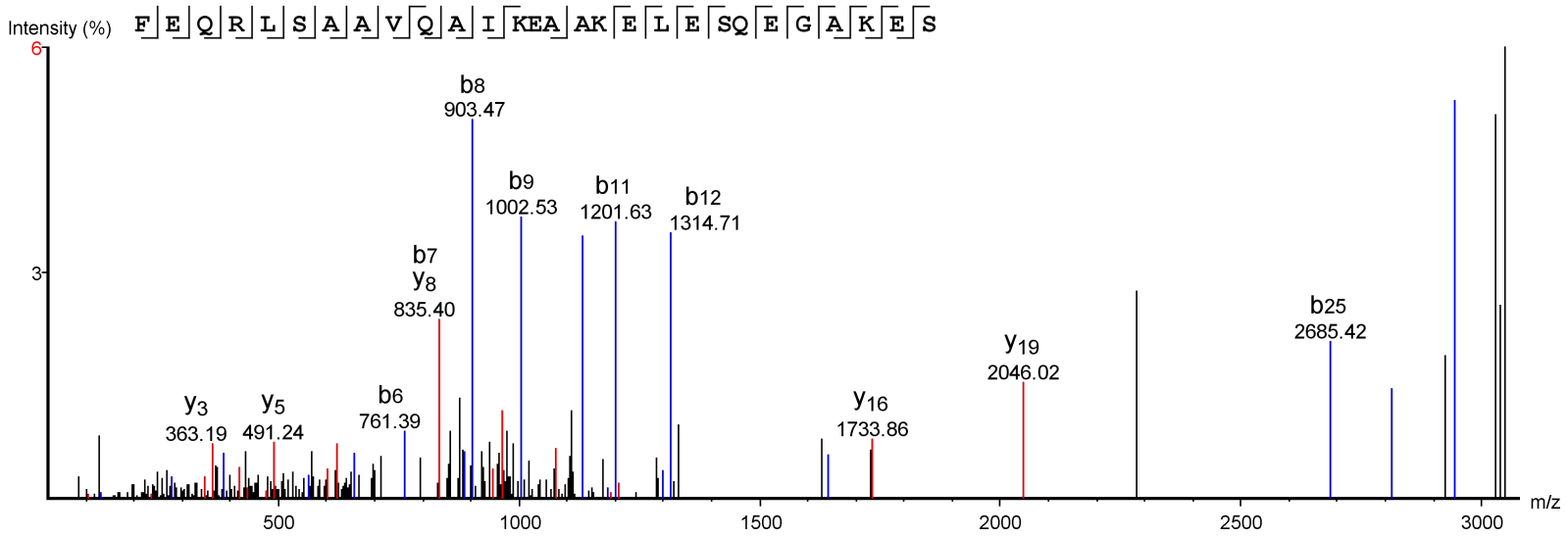


>Locus_39923_Transcript_1/2_Confidence_1.000_Length_940


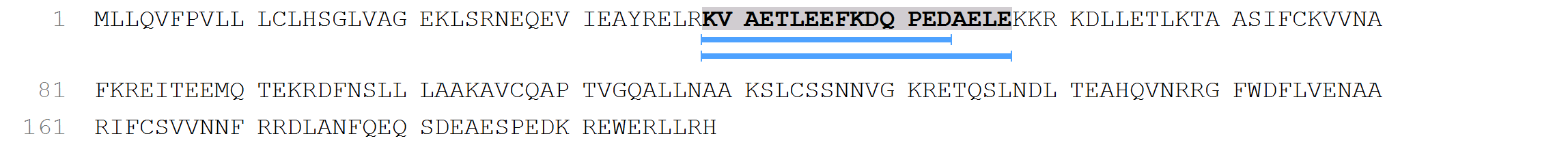


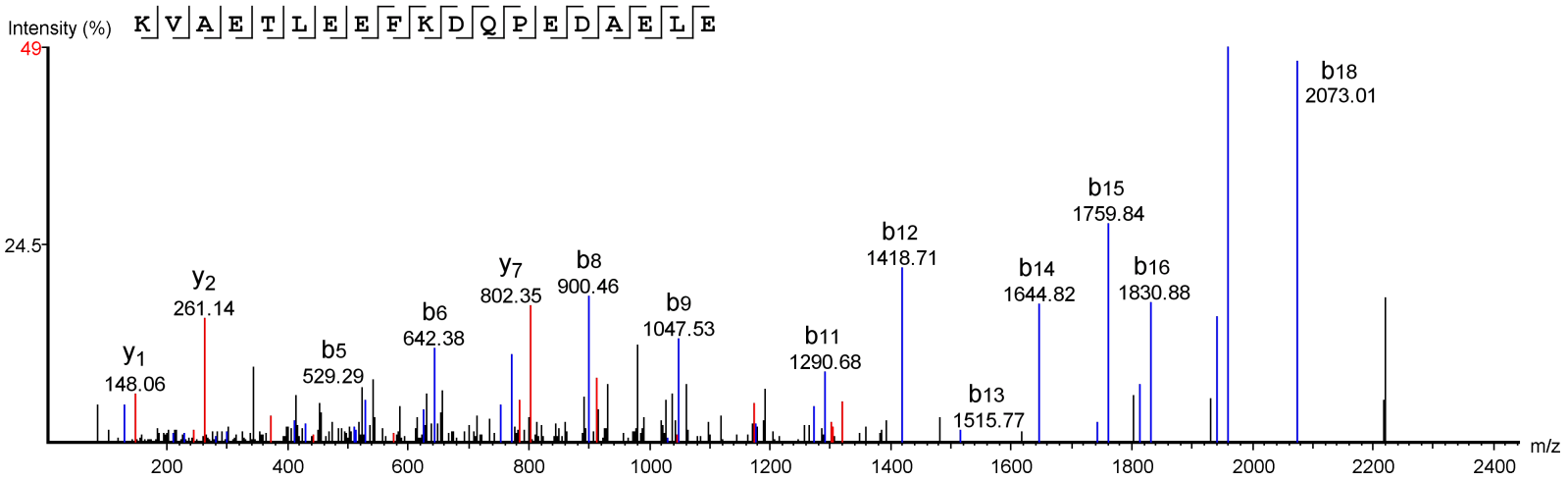


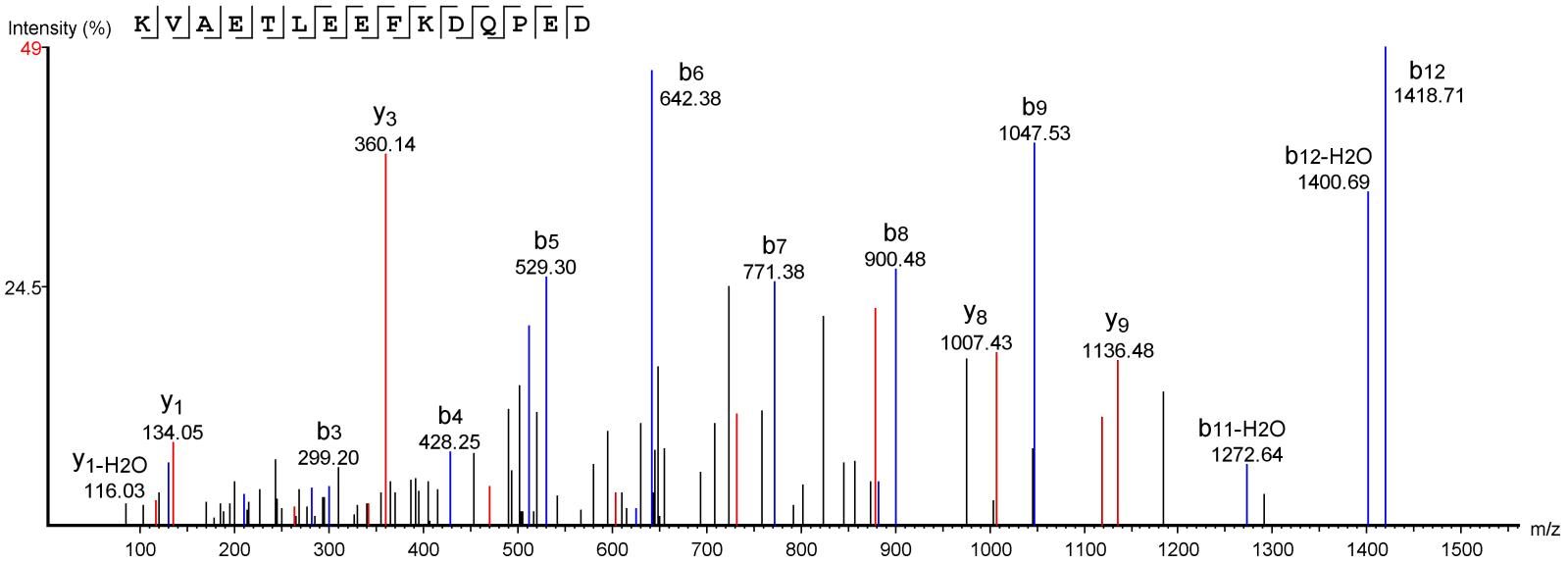


MLLQVFPVLLLCLHSGLVAGEKLSRNEQEVIEAYRELRKVAETLEEFKDQPEDAELEKKRKDLLETLKTAASIFCKVVNAFKREITEEMQTEKRDFNSLLLAAKAVCQAPTVGQALLNAAKSLCSSNNVGKRETQSLNDLTEAHQVNRRGFWDFLVENAARIFCSVVNNFRRDLANFQEQSDEAESPEDKREWERLLRH


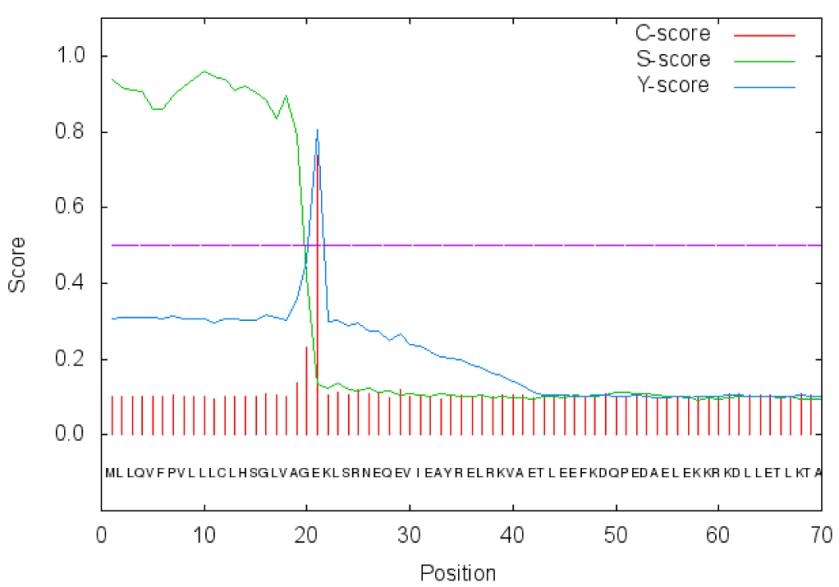


>Locus_12313_Transcript_2/2_Confidence_0.938_Length_821 +3 279 821


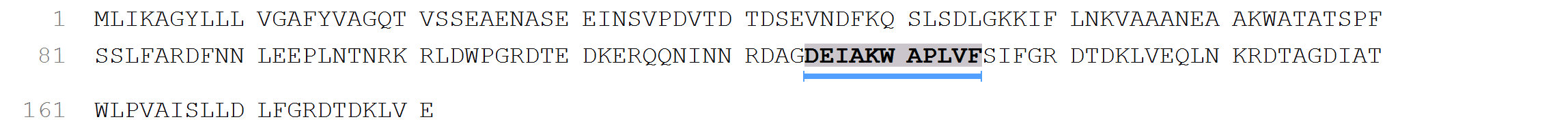


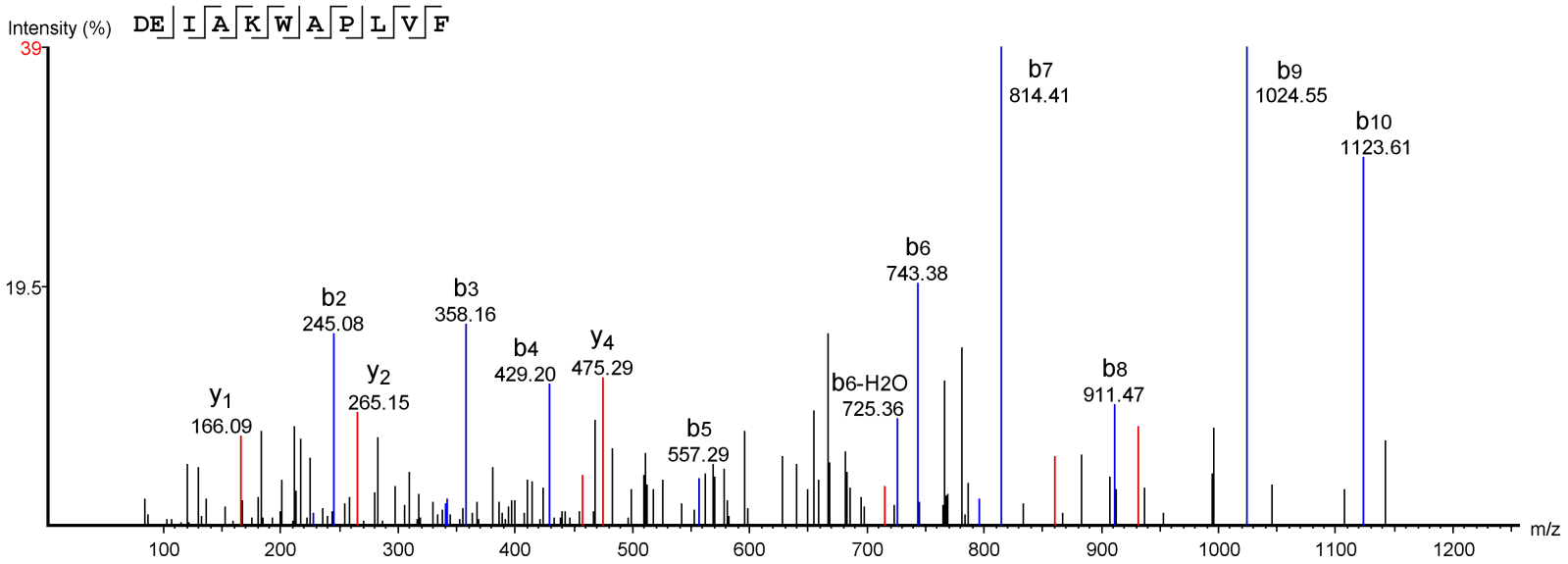


MLIKAGYLLLVGAFYVAGQTVSSEAENASEEINSVPDVTDTDSEVNDFKQSLSDLGKKIFLNKVAAANEAAKWATATSPFSSLFARDFNNLEEPLNTNRKRLDWPGRDTEDKERQQNINNRDAGDEIAKWAPLVFSIFGRDTDKLVEQLNKRDSDDGIATWLPIAISVLDLFGRDTDKLVE


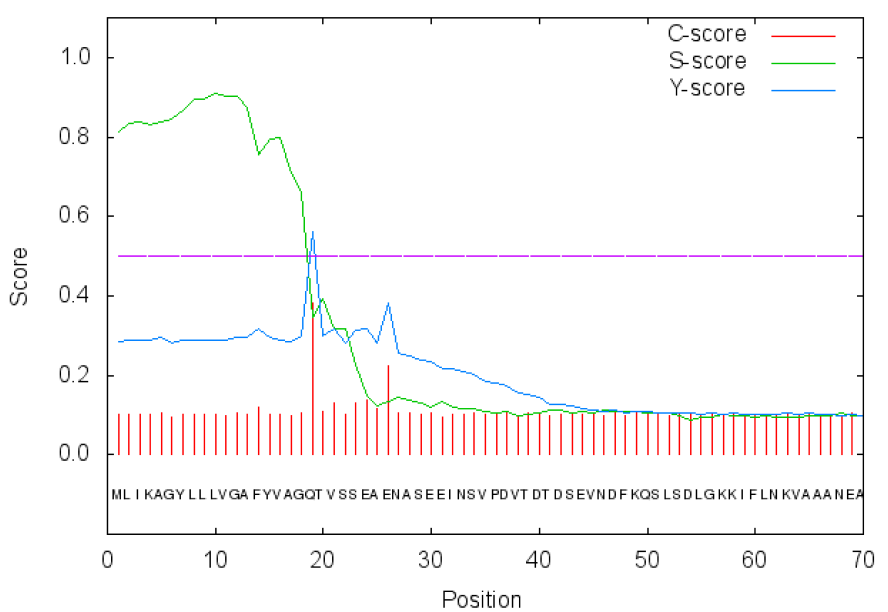


**B. Prediction (SignalP 4.1) of signal peptide of precursor proteins identified in fractions for LC-MS/MS**

>Locus_32929_Transcript_1/2_Confidence_1.000_Length_785 -2 2 550

MFLQVSFTFLLSLVLASALPTSDDEYQTLSRELQTLQQEFSKRDFWEGLWKAGSRICKIAKTLSLDNNADNANNANNDTEVDPVQVQKRDFWNNLLTVVNKVCKAASETERDITSGLDPEVADDKRDFWEDLGNVGKKVCQVADTLANNGRRSGEYVEQDPELRRKRFLWPDLVTTVDQICKAAGR


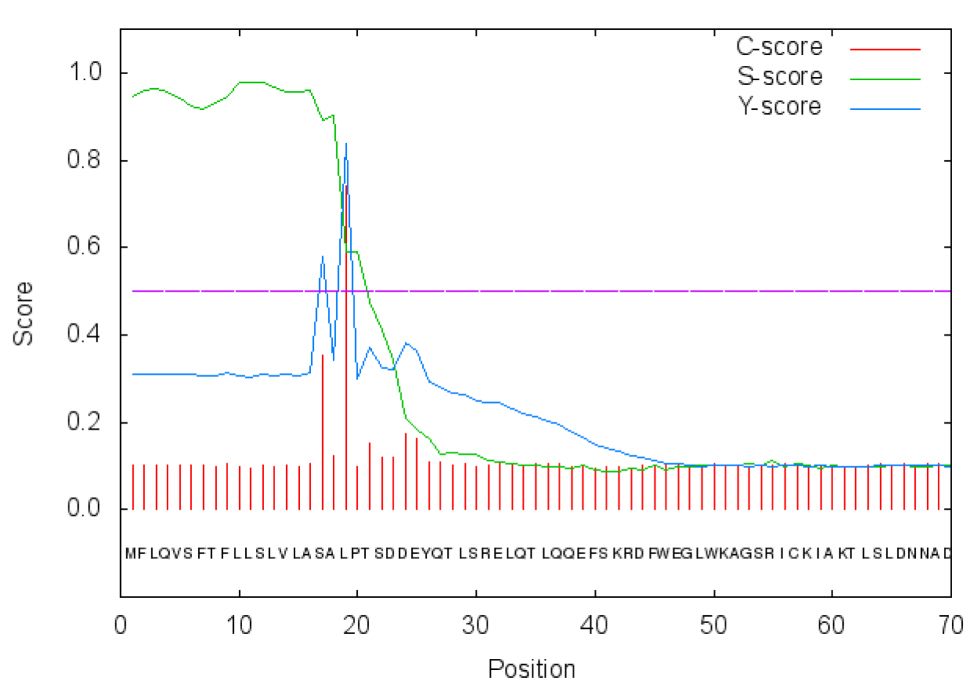

Supplement: S1 File — (DOCX) [file pntd.0006948.s007.docx]
